# Reliability of Theory of Mind Tasks in Schizophrenia, ASD, and Nonclinical Populations: A Systematic Review and Reliability Generalization Meta-analysis

**DOI:** 10.1007/s11065-024-09652-4

**Published:** 2024-10-08

**Authors:** Harry Kam Hung Tsui, Ting Yat Wong, Chak Fai Ma, Ting Eva Wong, Janet Hsiao, Sherry Kit Wa Chan

**Affiliations:** 1 Department of Psychiatry, Li Ka Shing Faculty of Medicine, The University of Hong Kong, Hong Kong SAR, China; 2 Department of Psychology, Education University of Hong Kong, Hong Kong SAR, China; 3 School of Nursing, The Hong Kong Polytechnic University, Hong Kong SAR, China; 4 Department of Psychology, The University of Hong Kong, Hong Kong SAR, China; 5 Division of Social Science, Hong Kong University of Science & Technology, Hong Kong SAR, China; 6 The State Key Laboratory of Brain and Cognitive Sciences, The University of Hong Kong, Hong Kong SAR, China

**Keywords:** Theory of mind, Mentalising, Psychometric reliability, Autism spectrum disorders, Schizophrenia, Meta-analysis, Systematic review

## Abstract

Though theory of mind (ToM) is an important area of study for different disciplines, however, the psychometric evaluations of ToM tasks have yielded inconsistent results across studies and populations, raising the concerns about the accuracy, consistency, and generalizability of these tasks. This systematic review and meta-analysis examined the psychometric reliability of 27 distinct ToM tasks across 90 studies involving 2771 schizophrenia (SZ), 690 autism spectrum disorder (ASD), and 15,599 nonclinical populations (NC). Findings revealed that while all ToM tasks exhibited satisfactory internal consistency in ASD and SZ, about half of them were not satisfactory in NC, including the commonly used Reading the Mind in the Eye Test and Hinting Task. Other than that, Reading the Mind in the Eye Test showed acceptable reliability across populations, whereas Hinting Task had poor test–retest reliability. Notably, only Faux Pas Test and Movie for the Assessment of Social Cognition had satisfactory reliability across populations albeit limited numbers of studies. However, only ten studies examined the psychometric properties of ToM tasks in ASD adults, warranting additional evaluations. The study offered practical implications for selecting ToM tasks in research and clinical settings, and underscored the importance of having a robust psychometric reliability in ToM tasks across populations.

**Supplementary Information:**

The online version contains supplementary material available at 10.1007/s11065-024-09652-4.

## Introduction

Theory of mind (ToM) refers to the ability to understand and infer other’s thoughts, beliefs, and behaviors and is a major domain of social cognition (Frith & Frith, 2005; Premack & Woodruff, 1978). Numerous ToM tasks have been developed and utilized across disciplines with some variations of ToM operationalizations, including reasoning false beliefs, recognizing a faux pas, interpreting humors or lies, and inferring mental states and intentions from cartoons and 2D geometric shapes. However, psychometric properties of these ToM tasks, particularly their reliability, were often inconsistent and underexplored in specific clinical and nonclinical populations, which is fundamental for ensuring accurate and generalizable clinical and research applications (Beaudoin et al., 2020; Gourlay et al., 2020; Osterhaus & Bosacki, 2022; Yeh et al., 2021; Yeung et al., 2024).

The impairment of ToM has been suggested to be one of the core features in schizophrenia (SZ) (Bora et al., 2009; Sprong et al., 2007) and autism spectrum disorder (ASD) (Frith & Happé, 1994), but has also displayed a transdiagnostic nature in other conditions, such as major depressive disorder, anxiety disorder, and borderline personality disorder (Bora & Berk, 2016; Cotter et al., 2018; Németh et al., 2018; Plana et al., 2014). Accurately attributing others’ mental states is vital to effectively navigate daily social interactions, and thus the impairment of ToM is linked to poor psychosocial functioning including communication and interpersonal difficulties (Couture et al., 2011; Happé, 2015). Meta-analyses and reviews (Chung et al., 2014; Fernandes et al., 2018; Oliver et al., 2021; Veddum & Bliksted, 2022) have provided evidence of similar levels of ToM impairments between ASD and SZ. Nonetheless, ToM tasks have presented varying psychometric properties in ASD, SZ, and nonclinical populations (e.g., Gourlay et al., 2020; Morrison et al., 2019; Pinkham et al., 2018), indicating that robustness of the psychometric properties of tasks might vary across the tested populations. These led to the concerns of the validity and reliability of ToM tasks to measure their intended construct in clinical and nonclinical populations, and thus questioning on the interpretation and comparison of ToM assessments (Gernsbacher & Yergeau, 2019; Konstantin et al., 2023; Quesque & Rossetti, 2020).

Psychometric reliability of psychological assessments is one of the keys of quantitative research and a prerequisite of validity in a reflective model, which indicates an association between ability and task score (Bollen & Lennox, 1991; DeVellis, 2006; Roberts & Priest, 2006). Classical test theory provides a conceptual and practical basis for many measurement tools including ToM tasks, which posits that the observed score is the sum of the true score and the error score (DeVellis, 2006; Fu et al., 2023). In classical test theory, reliability refers to the consistency, stability, and repeatability of a test or measurement, which is defined as the proportion of the variance in observed test scores that reflects true variance in the underlying trait or ability being measured, as opposed to variance caused by measurement error. One of the reliability estimates is internal consistency, which refers to the consistency among items to measure the same underlying construct. Poor internal consistency suggests imprecise measurement of the intended construct and obscures the true effect size. However, while internal consistency measured by Cronbach’s alpha has been widely used, it should be noted that it has been considered to have major statistical flaws and was not regarded as a useful guidance for reliability (Sijtsma, 2009). Test–retest reliability indicates the stability of results of the same individual over time and the potential influences of the random errors. Inter-rater reliability refers to the degree of agreement among different raters scoring the same task. ToM tasks with satisfactory reliability are crucial to accurately investigate differences between individuals, between groups, and over time in clinical and research contexts (Davidson et al., 2018; Osterhaus & Bosacki, 2022). Reviews and large-scale research have examined the psychometric properties of ToM tasks in children and adolescents (Ahmadi et al., 2015; Beaudoin et al., 2020; Fu et al., 2023; Hayward & Homer, 2017; Poll et al., 2023), adults (Gourlay et al., 2020; Klein et al., 2022 (SCOPE); Yeung et al., 2024), individuals with SZ (Davidson et al., 2018; Pinkham et al., 2014 (SCOPE); Yeh et al., 2021), ASD (Morrison et al., 2019 (SCOPE)), and other neuropsychiatric populations (Eddy, 2019). Results from the large-scale SCOPE project have recommended the use of Hinting Task for SZ and ASD, as well as The Awareness of Social Inference Test—Part three for ASD, but no ToM tasks were recommended for the nonclinical population based on the psychometric properties. Meanwhile, Gourlay et al. (2020) suggested that the modified version of the Strange Stories Task for the healthy population across ages had satisfactory psychometric properties. However, these studies have evaluated a limited scope of ToM tasks, and no meta-analysis of psychometric reliability of ToM tasks has been conducted. Studies also have indicated different psychometric properties of the same task between clinical and nonclinical populations (Morrison et al., 2019; Pinkham et al., 2018). Particularly, the same ToM tasks used in nonclinical populations tended to have poorer reliability than that in the clinical populations, including Reading the Mind in the Eye Test, Hinting Task, The Awareness of Social Inference Test—Part three, and Social Attribution Task-multiple choice. On the other hand, a recent review and meta-analysis by Kittel et al. (2022) focusing on internal consistency of Reading the Mind in the Eye Test suggested an acceptable level of reliability (Cronbach’s alpha = 0.73) across multiple populations, while the proportion of participants with a clinical diagnosis was not a significant moderator. Different scopes of psychometric properties were assessed in these studies which may explain the differences in the conclusion. Furthermore, different study methodologies may also explain the different findings. Only moderation effect of proportion of different study populations on internal consistency was examined in the Kittel et al (2022), potentially over-generalizing results of studies mixing clinical and nonclinical populations (Kittel et al., 2022). Therefore, a comprehensive quantitative examination on psychometric reliability of ToM tasks in clinical and nonclinical populations to understand the accuracy, consistency, reproducibility, and generalizability of different ToM tasks would be essential for further research and clinical applications.

The current study aimed to conduct a systematic review and meta-analysis of the psychometric reliability of ToM tasks in SZ, ASD, and nonclinical populations. These include internal consistency, test–retest reliability of ToM tasks, and inter-rater reliability for those that require multiple raters. The moderating effects of demographics, study designs, and task scores on the reliability would also be explored. Although Reading the Mind in the Eye Test has been suggested to be more closely related to emotion recognition rather than ToM (Kittel et al., 2022; Oakley et al., 2016; Quesque & Rossetti, 2020), this task was still included due to its pervasive usage in the field. This study would provide valuable insights on the psychometric properties of ToM tasks and practical guidance for the future study of ToM in clinical and nonclinical populations.

## Method

### Search Strategy

A systematic search of the literature was conducted using the following electronic databases: EMBASE (1947–2023), MEDLINE (1946–2023), PubMed (1843–2023), Web of Science (1956–2023), and PsycINFO (1806–2023). The initial keywords used were the following: theory of mind OR perspective taking OR mind-reading OR mental representation OR mind understanding OR mentaliz* AND test–retest reliability OR test–retest correlation OR intraclass correlation OR internal consistency OR internal reliability OR inter-rater reliability OR inter-rater consistency AND general population OR healthy population OR healthy adults OR community sample OR non-clinical sample OR schizophrenia OR psychosis OR autism OR ASD. Literature searches were conducted by two independent researchers (H.K.H.T. and T.E.W.) from database inception until September 8, 2023, and the search was conducted between September 8, 2023, and September 14, 2023. References were integrated into a software reference manager, EndNote 20, for deduplication and screening. The current meta-analysis was registered on PROSPERO (Supplementary Material 3 for de-identified protocol) and was conducted based on the Preferred Reporting Items for Systematic Reviews and Meta-analyses (PRISMA) guidelines (Page et al., 2021) and the Reliability Generalization Meta-analysis (REGEMA) checklist (Sánchez-Meca et al. 2021) (Supplementary Material 1 and 2). The inter-rater agreements measured by kappa coefficients of title/abstract screening and full-text screening were 0.804 and 0.815, respectively, indicating excellent agreement between the raters during study selection.

### Eligibility Criteria

Studies that fulfilled the following criteria were included in the current study: (1) included at least one ToM task defined by the authors; (2) reported internal consistency (mainly Cronbach’s alpha), test–retest reliability, or inter-rater reliability (e.g., intraclass correlation (ICC) coefficients, Pearson’s correlations, kappa coefficient), or inferential statistical data for calculation; (3) published in English language; (4) published as original peer-reviewed research articles; (5) involved targeted samples aged 16 or above who were from one of the targeted populations, schizophrenia (SZ), ASD, or nonclinical (NC) populations. The NC population refers to individuals without psychiatric diagnoses. Studies were excluded if the study population was below the age of 16; were reviews, qualitative studies, clinical cases, abstracts, protocols, or conference posters; or did not report meta-analyzable data or were unable to be obtained from the authors.

### Data Extraction

Information of each study was extracted by two researchers (H.K.H.T. and T.E.W.) independently and was cross-checked with disagreements aligned through consensus meetings with the research team. The inter-rater agreement of data extraction measured by ICC was 0.983. Information about the details of the ToM measurements and the psychometric reliability were recorded. These included internal consistency, test–retest reliability, inter-rater reliability, retest intervals, the name of tasks, and the general description of the tasks. For each ToM task assessed in the included studies, only one internal consistency, test–retest reliability, and inter-rater reliability from each population was extracted. In instances where studies reported results from longitudinal research, we only selected the baseline psychometric properties. Therefore, no reliability estimates were taken from the same sample repeatedly for each ToM task for the same population to ensure the independence of the effect sizes. Demographic details of the sample, including age, gender, years of education, diagnoses for clinical populations, and other important study characteristics including sample size, published year, and country of the study, were also recorded. The construct validity of tasks was also preliminarily evaluated using the mentalizing and nonmerging criteria proposed by Quesque and Rossetti (2020). The mentalizing criterion evaluates whether the success of participants in completing a task can be attributed to their ability to understand and infer mental states, instead of lower-level processes such as associative learning. The nonmerging criterion examines whether the task requires participants to represent others’ mental state, distinguishing between their personal mental states and those of others.

### Risk of Bias Assessment

Risk of bias assessment was conducted by two researchers (H.K.H.T. and J.L.) independently to evaluate the quality of included studies using the Consensus-based Standards for the selection of health status Measurement Instruments (COSMIN) checklist items guidelines (Mokkink et al., 2010) (Supplementary Table 5). Studies were categorized into four levels of quality: very good, adequate, doubtful, and inadequate. Disagreements were resolved through consensus meetings with the research teams. The inter-rater agreement measured by kappa coefficients was 0.826.

### Statistical Analysis

This review presents meta-analytic results (*k* ≥ 2) where possible and reports individual study findings (*k* = 1) descriptively to provide a comprehensive literature overview of the psychometric reliability of ToM tasks. Reliability estimates were calculated using Fisher’s *z* for test–retest reliability and internal consistency (Sánchez-Meca et al., 2013). To enhance normality for analysis, internal consistency measured with Cronbach’s alpha was square-root before Fisher’s *z* transformation (Bonett, 2010; Thompson & Vacha-Haase, 2000). Random-effects meta-analytic model with restricted maximum likelihood (REML) estimator and inverse-variance weighting was implemented to account for between-study variance and examine the pooled reliability estimates of ToM tasks. Knapp and Hartung adjustment (Knapp & Hartung, 2003) was applied to account for uncertainty and potential biases in the standard errors due to the pooling of effect sizes. We examine ToM tasks with different populations, including NC, SZ, ASD, SZ mixed with NC (SZ-NC), and ASD mixed with NC (ASD-NC) to ensure homogeneity and generalizability to different study populations. Including mixed populations is essential to provide a comprehensive review that reflects the current research landscape as many studies indeed include a population with mixed diagnoses. This approach might also be beneficial for research examining the continuum from nonclinical to clinical populations, such as those within the autism spectrum and the psychosis spectrum, providing insights into how reliability measures perform across this spectrum. *I*^2^ value, Q statistic, and tau-squared (τ^2^) with REML estimation were used to evaluate the heterogeneity of the pooled reliability estimates. Potential publication biases were investigated using funnel plots, Egger’s test, and trim-and-fill procedure. For ease of interpretation, the reliability estimates have been converted back into their original coefficients and are reported as such. To meaningfully analyze reliability estimates of ToM tasks, only tasks that were identical or equivalent in method, instrument, and format were pooled together for analysis without including any modified or shortened version. Pearson’s correlation (*r*) and intraclass correlation (ICC) in test–retest reliability were analyzed separately due to their differences in statistical calculation. For the general rules of thumb, internal consistency of 0.70 is considered satisfactory in the early stage of research but a level of 0.80 or higher is preferred for rigorous clinical and experimental settings (Lance et al., 2006; Nunnally & Bernstein, 1994). For test–retest reliability and inter-rater reliability, satisfactory cut-off values were established as follows: 0.75 or above for ICC and Cohen’s Kappa, and 0.70 or above for correlation coefficients (Cicchetti, 1994; Fleiss, 1986). Additionally, univariate meta-regressions of internal consistency and test–retest reliability were conducted with mean age, gender (male proportion), years of education, sample size, mean and standard deviation of task scores, continent of the study, quality category from the risk of bias assessment, and publication years of studies in different populations as moderators. Exploratory meta-regression analyses were performed depending on the availability of data (≥ 4 estimates) using random-effects models which assume that the true effect sizes vary across studies. Additional meta-regression was also conducted to directly compare between study populations, when there were three or more estimates in two or more populations within a task. The random-effects model accounts for both sampling error within each study and differences between studies, allowing us to explore the impact of study-level factors on the effect sizes, while considering the inherent diversity of the included studies. Besides, as inter-rater reliability was only applicable to tasks that involve raters and relevant studies were limited, it was only included in the systematic review. Only ToM tasks that have more than four studies provided psychometric reports were included in the main analyses to provide a robust evaluation of the psychometric reliability. Having a *p* value less than 0.05 is considered statistically significant in all tests. Statistical analysis was conducted with the package metafor (version 3.8–1) in R version 4.2.1. The data file and analysis code for this study are openly available in the Open Science Framework (OSF) repository at https://osf.io/sj746/?view_only=68c65ff7db8541edb3c952699e4e8d7f.

## Results

### Database Characteristics

**Fig. 1:**
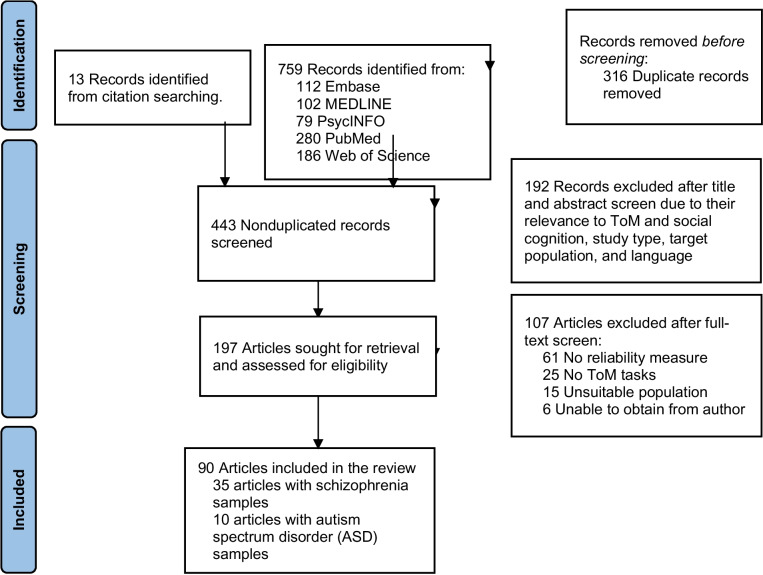
Preferred Reporting Items for Systematic Reviews and Meta-Analyses (PRISMA) flow diagram showing study selection. Literature searches were conducted by two independent researchers (K.T & E.W) from database inception until September 8, 2023. ToM indicates theory of mind. *From:* Page MJ, McKenzie JE, Bossuyt PM, Boutron I, Hoffmann TC, Mulrow CD, et al. The PRISMA 2020 statement: an updated guideline for reporting systematic reviews. BMJ 2021;372:n71. 10.1136/bmj.n71. For more information, visit: http://www.prisma-statement.org/

Of the 759 reports screened, 90 studies met the inclusion criteria of this review with 35 studies involving SZ samples (12 studies were SZ-NC) and 10 studies involving ASD samples (5 studies were ASD-NC) (Fig. 1 and Supplementary Table 2). The quality assessment of included study using the COSMIN guideline indicated that 36 (40%) studies had very good quality, 39 (43.3%) studies were adequate in quality, and 15 (16.7%) studies had doubtful quality (Supplementary Table 5). A summary of the risk-of-bias evaluation was reported in Supplementary Fig. 1. The total sample sizes of populations were 15,599 NC (mean age = 30.60; 50.1% male), 2771 SZ (mean age = 37.57; 58.2% male), and 690 ASD (mean age = 28.73; 74.6% male). This review obtained 128 internal consistency estimates, 66 test–retest reliability estimates, and 16 inter-rater reliability estimates. Seventy-six studies reported internal consistency of ToM tasks where 28 and 7 studies involved SZ and ASD, respectively. For test–retest reliability, there were twenty-six studies with 14 conducted in SZ samples and only 2 studies for the ASD population. The range and median of intervals between test and retest were 7–365 and 21 days for NC and 14–360 and 17 days for SZ. Twenty-seven distinct ToM tasks in the current review were categorized by operationalizations and assessed with the construct validity, and their psychometric reliability was evaluated with the rules of thumb by populations (Table 1). Reading the Mind in the Eye Test (41 studies), Hinting Task (23), The Awareness of Social Inference Test—Part three (9), Faux Pas Test (8), the Short Stories Task (5), Social Attribution Task-multiple choice (5), Picture Sequencing Task (4), and Movie for the Assessment of Social Cognition (4) were the eight ToM tasks with four or more studies reported psychometric reliability, and the meta-analyses were thus focused on these tasks for the sake of conciseness and interpretability (Table 2). ToM tasks with less than four studies were shown in the supplementary materials (Supplementary Table 1). Internal consistency and test–retest reliability of these eight ToM tasks were summarized in Table 3.

**Table 1:** Summary of included ToM tasks categorized by operationalization

Operationalization	Task	*n*	Internal consistency	Test–retest reliability	Inter-rater reliability	Mentalizing criteria/ nonmerging criteria^a^
Interpreting intentions in non-direct speech	Hinting Task (HT) (Corcoran et al., 1995)	23	**SZ: 0.71 (13)** **ASD: 0.77 (2)** NC: 0.55 (8)	SZ: 0.65 (5) NC: 0.53 (3)	**SZ: 0.85 (1)** **NC: 0.83 (1)**	Yes/Yes
	Strange Stories (Happé, 1994)	2	SZ: 0.50 (1)			Yes/Yes
	Strange Stories-revised (White et al., 2009)	1		NC: 0.63 (1)	**NC: 0.93 (1)**	Yes/Yes
	Social Knowledge Test (SKT) (Achim et al., 2012)	1		**NC: 0.77 (1)**	**NC: 0.99 (1)**	Yes/Yes
Reasoning false belief	The Short Story Task (TSST) (Dodell-Feder et al., 2013)	5	**ASD-NC: 0.73 (1)** NC: 0.66 (4)		**ASD-NC: 0.80 (1)** **NC: 0.98 (1)**	Yes/Yes
Spotting a faux pas	Faux Pas Test (FPT) (Stone et al., 1998)	8	**NC: 0.89 (5)**	**SZ: 0.76 [0.61–0.86] (1)**	**SZ-NC: 0.76 (1)**	Yes/Yes
	FPT-short version (Şandor & İşcen, 2023)	3	**SZ: 0.86 (2)** **NC: 0.95 (2)**	**SZ: 0.78 (1)** **NC: 0.97 (1)**	**SZ: 0.82 (1)** **NC: 0.87 (1)**	Yes/Yes
Inferring intentions and interactions of individuals in a naturalistic video	The Awareness of Social Inference Test—Part three (TASIT-III) (Lim et al., 2020)	9	**SZ: 0.80 (5)** **ASD: 0.86 (1)** **NC: 0.73 (5)**	SZ: 0.62 (5) NC: 0.62 (3)		Yes/Yes
	Movie for the Assessment of Social Cognition (MASC) (Dziobek et al., 2006)	4	**SZ-NC: 0.87 (1)** **ASD-NC: 0.85 (2)** **NC: 0.78 (2)**	**SZ-NC: 0.85 (1)** **ASD: 0.92 (1)** **NC: 0.89 (1)**	**ASD: 0.98 (1)** **NC: 0.94 (1)**	Yes/Yes
	Strange Stories film task (SSFT) (Murray et al., 2017)	2	ASD: 0.62 (1)			Yes/Yes
	Adult-Theory of Mind (A-ToM) (Brewer et al., 2017)	1	**ASD: 0.82 (1)**	**ASD: 0.82 (1)**	**ASD: 0.77 (1)**	Yes/Yes
	Assessment of ToM for people with Schizophrenia (AToMS) (Yeh et al., 2023)	1	**SZ: 0.85 (1)**	**SZ: 0.90 (1)**	**SZ: 0.99 (1)**	Yes/Yes
	Battery for the Assessment of ToM (BAT) (Serra-Mayoral et al., 2021)	1	**SZ-NC: 0.89 (1)**			Yes/Yes
	The Virtual Assessment of Mentalising Ability (VAMA) (Canty et al., 2017)	1	NC: 0.69–0.84 (1)	**NC: 0.95–0.99 (1)**		Yes/Yes
	Versailles-Situational Intention Reading (V-SIR) (Bazin et al., 2009)	1	**NC: 0.74 (1)**			Yes/Yes
Inferring intentions and/or interactions from cartoons or comics	Picture Sequencing Task (PST) (Brüne, 2003)	5	**SZ: 0.84 (2)** **ASD: 0.72 (1)** NC: 0.64 (2)	SZ: 0.59 (2)		Yes/No
	PST-modified (Zıvralı Yarar et al., 2021)	1	**ASD-NC: 0.71–0.78 (1)**			Yes/No
	Comic Strip Task (CST) 14-item (Le Donne et al., 2023)	1	**NC: 0.70 (1)**			Yes/No
	CST 34-item (Brunet et al., 2003)	2	**SZ: 0.74 (1)**	SZ: 0.71 (1)		Yes/No
	CST 48-item (Lee et al., 2010)	1	**SZ-NC: 0.84 (1)**	SZ-NC: 0.57 (1)		Yes/No
	The Humor Comprehension and Appreciation Test (ToM-HCAT) (Aykan & Nalçacı, 2018)	1	**NC: 0.94 (1)**			Yes/No
	Yoni Task 48-item (Shamay-Tsoory & Aharon-Peretz, 2007)	1	**NC: 0.90 (1)**			Yes/No
Inferring intentions and interactions of 2D geometric shapes	Social Attribution Task-multiple choice (SAT-MC) (Bell et al., 2010)	5	**SZ: 0.82 (3)** **NC: 0.72 (3)**	SZ: 0.64 (2) NC: 0.55 (1)		No/No
	Social shape task (SST) (Brown et al., 2019)	2	**ASD: 0.72 (1)** NC: 0.67 (2)			No/No
	Animated Triangles Task-MC (White et al., 2011)	2	NC: 0.62 (2)			No/No
	Animated Triangles Task-verbal (Andersen et al., 2022)	2	**ASD-NC: 0.72 (1)** NC: 0.54 (1)	**NC: 0.77–0.97 (1)**	**ASD-NC: 0.87 (1)**	No/No
Inferring mental states in facial expressions	Reading the Mind in the Eyes Test (RMET) (Baron-Cohen et al., 2001)	41	**SZ: 0.72 (3)** **ASD: 0.77 (1)** NC: 0.65 (31)	**SZ: 0.73 (4)** **NC: 0.76 (7)**		No/No
Multiple operations	The Combined Stories Test (COST) (Achim et al., 2012)	2	**SZ-NC: 0.81 (1)**	**NC: 0.84 (1)**	**SZ-NC: 0.98 (1)** **NC: 0.75 (1)**	Yes/Yes

Note: ASD indicates autism spectrum disorder; *SZ*, schizophrenia; *NC*, nonclinical population; *ASD-NC*, autism spectrum disorder and nonclinical population mixed together; *SZ-NC*, schizophrenia and nonclinical population mixed together. Numbers in brackets after the reliability estimates indicate the number of studies reported. *n* indicates the number of studies that involved the specific ToM task. For internal consistency, values with 0.70 or above were bolded. For test–retest reliability and inter-rater reliability, ICC and Cohen’s kappa values with 0.75 or above were bolded, and correlation coefficients with 0.70 were bolded (Cicchetti, 1994; Fleiss, 1986). The superscript “^a^” indicates whether the task met the mentalizing criteria and non-merging criteria for construct validity as suggested by Quesque and Rossetti (2020)

**Table 2:** Demographics, sample size, and reported reliability estimates of the ToM tasks with four or more psychometric studies

Task	Population	*k*	*n* in total (*n* of HC)	Age, mean	Gender (male %)	Education, mean	No. IC reported (Cronbach’s alpha)	No. TRR reported (Pearson’s *r*)	No. IRR reported
RMET	NC	36	8752	28.29	48.6	13.31	31	12 (7 ICC)	
	SZ	6	536	40.12	70.5	13.01	3	5 (1 ICC)	
	SZ-NC	1	70 (40)	37.9, 35.5	36.7, 8.5	12.9, 13.9	2	1 (ICC)	
	ASD	1	103	24.28	89.3	NR	1		
	ASD-NC	1	37 (19)	21.87, 22.90	93.78, 95.24	11.91, 12.71	1		
HT	NC	8	1153	33.74	57.1	14.68	8	6 (1 ICC, 1 Kappa)	1
	SZ	14	1664	37.69	64.3	12.95	13	7 (1 ICC, 1 Kappa)	1
	SZ-NC	5	515 (244)	35.68, 33.50	65.2, 63.3	11.70, 12.26	5		
	ASD	2	131	25.14	87.5	NR	2		
TASIT	NC	5	631	31.78	58.1	14.77	5	5 (1 ICC)	
	SZ	7	685	37.13	66.5	13.37	5	6 (1 ICC)	
	ASD	1	103	24.28	89.3	NR	1		
FPT	NC	6	1088	32.53	37.0	13.18	7	1	2
	SZ	3	137	40.86	63.0	NR	1		
	SZ-NC	1	71 (31)	30.20, 29.97	45.0, 29.0	10.65, 11.74		1	1
MASC	NC	4	612	28.18	58.1	15.65	2	1 (ICC)	1
	ASD-NC	2	87 (46)	31.77, 31.41	88.42, 77.69	15.6, 15.65	2	1 (ICC)	1
	SZ-NC	1	167 (123)	47.61, 26.05	56.1, 30.2	NR	1	1 (ICC)	
SAT-MC	NC	3	265	31.07	51.3	14.52	3	1	
	SZ	3	310	41.42	57.9	12.79	3	2	
	SZ-NC	1	151 (85)	NR	NR	NR	1		
TSST	NC	4	493	28.86	34.55	15.7	4		2
	ASD-NC	1	64 (32)	30.34, 31.13	62.5, 53.10	13.09, 14.64	1		1
PST	NC	2	295	28.59	63.0	11.9	2		
	ASD	1	103	24.28	89.3	NR	1		
	SZ	3	171	43.52	57.7	NR	2	2	

Note: ASD indicates autism spectrum disorder; *SZ*, schizophrenia; *NC*, nonclinical population; *ASD-NC*, autism spectrum disorder and nonclinical population mixed together; *SZ-NC*, schizophrenia and nonclinical population mixed together; *TRR*, test–retest reliability; *IC*, internal consistency; *IRR*, inter-rater reliability; *ICC*, intraclass correlation; *NR*, not reported; *RMET*, Reading the Mind in the Eye Test; *HT*, Hinting Task; *TASIT-III*, The Awareness of Social Inference Test—Part three; *FPT*, Faux Pas Test; *SAT-MC*, Social Attribution Task-multiple choice; *TSST*, The Short Story Task; *MASC*, Movie for the Assessment of Social Cognition

**Table 3:** Summary of internal consistency and test–retest reliability of ToM tasks from meta-analysis and systematic review

	Internal consistency (Cronbach’s alpha)			Test–retest reliability (Pearson’s r, if not specified)		
	SZ	ASD	NC	SZ	ASD	NC
RMET	0.694 [0.314–0.889] (3) **0.722 [0.666–0.771]** (3; SZ-NC)	**0.766 [0.688–0.827]** (1)	0.650 [0.604–0.692] (31)	**0.731 [0.537–0.851]** (4)	NR	**0.713 [0.634–0.778]** (5) **0.762 [0.652–0.848]** (7; ICC)
HT	**0.713 [0.655–0.763]** (13) 0.679 [0.633–0.720] (6; SZ-NC)	**0.774 [− 0.042–0.989]** (2)	0.554 [0.465–0.634] (8)	0.652 [0.587–0.709] (5)	NR	0.528 [0.404–0.633] (4)
TASIT-III	**0.798 [0.766–0.826]** (5)	**0.86 [0.80–0.90]** (1)	**0.732 [0.636–0.807]** (5)	0.621 [0.454–0.746] (5)	NR	0.624 [0.081–0.881] (3)
FPT	NR	NR	**0.890 [0.734–0.957]** (6)	**0.76 [0.61–0.86]** (1) **0.83** (1; SZ-NC)	NR	NR
SAT-MC	**0.817 [0.743–0.871]** (3) **0.83** (1; SZ-NC)	NR	**0.719 [0.323–0.906]** (3)	0.636 [-0.776–0.988] (2)	NR	0.554 (1)
TSST	NR	**0.73** (ASD-NC; 1)	0.659 [0.579–0.728] (4)	NR	NR	NR
MASC	**0.87** (1; SZ-NC)	**0.851 [0.669–0.938]** (2; ASD-NC)	**0.776 [0.398–0.931]** (2)	**0.85** (1; SZ-NC)	**0.92** (1)	**0.89** (1)
PST	**0.839 [0.117–0.984]** (2)	**0.72** (1)	0.642 [− 0.843–0.998] (2)	0.586 [-0.197–0.913] (2)	NR	NR

Note: Numbers in bracket after the reliability estimates indicate the number of studies reported. *ASD* indicates autism spectrum disorder; *SZ*, schizophrenia; *NC*, nonclinical population; *ASD-NC*, autism spectrum disorder and nonclinical population mixed together; *SZ-NC*, schizophrenia and nonclinical population mixed together; *RMET*, Reading the Mind in the Eye Test; *HT*, Hinting Task; *TASIT-III*, The Awareness of Social Inference Test—Part three; *FPT*, Faux Pas Test; *SAT-MC*, Social Attribution Task-multiple choice; *TSST*, The Short Story Task; *MASC*, Movie for the Assessment of Social Cognition. Bold indicates a satisfactory or above (> 0.70) reliability

### Internal Consistency

Results indicated that the Cronbach’s alpha of Reading the Mind in the Eye Test and Hinting Task was 0.766 [0.688–0.827] (*k* = 1) and 0.774 [0.658–0.855] (*k* = 2, *α* = , *I*^2^ = 32.70%, Tau^2^ = 0.012) in ASD, respectively (Tables 3 and 4). Internal consistency of The Awareness of Social Inference Test—Part three and Movie for the Assessment of Social Cognition was acceptable in ASD (*k* = 1, *α* = 0.86 [0.80–0.90]) and ASD-NC (*k* = 2, *α* = 0.851 [0.780–0.901], *I*^2^ = 0.00%, Tau^2^ = 0.010), respectively. The Cronbach’s alpha of the Short Stories Task was 0.73 (*k* = 1) in ASD-NC. In the SZ population, internal consistency of Faux Pas Test was 0.933 (*k* = 1). The Cronbach’s alpha was 0.839 [0.117–0.984] (*k* = 2, *I*^2^ = 4.77%, Tau^2^ = 0.001) for Picture Sequencing Task, 0.798 [0.784–0.810] (*k* = 5, *I*^2^ = 0.00%, Tau^2^ = 0.000) for The Awareness of Social Inference Test—Part three, and 0.817 [0.743–0.871] (*k* = 3, *I*^2^ = 0.00%, Tau^2^ = 0.000) for Social Attribution Task-multiple choice in SZ, and the Cronbach’s alpha of Movie for the Assessment of Social Cognition was 0.87 (*k* = 1) in SZ-NC. Internal consistency of Reading the Mind in the Eye Test was 0.694 [0.314–0.889] in SZ (*k* = 3, *I*^2^ = 79.55%, Tau^2^ = 0.032) and 0.722 [0.666–0.771] (*k* = 3, *I*^2^ = 0.00%, Tau^2^ = 0.000) in SZ-NC, whereas that of Hinting Task was 0.713 [0.655–0.763] in SZ (*k* = 13, *I*^2^ = 74.62%, Tau^2^ = 0.026) and 0.679 [0.633–0.720] in SZ-NC (*k* = 6, *I*^2^ = 0.00%, Tau^2^ = 0.000). For the NC population, internal consistency was 0.890 [0.734–0.957] (*k* = 5, *I*^2^ = 96.03%, Tau^2^ = 0.148) for Faux Pas Test, 0.732 [0.600–0.827] (*k* = 5, *I*^2^ = 78.82%, Tau^2^ = 0.034) for The Awareness of Social Inference Test—Part three, 0.719 [0.323–0.906] (*k* = 3, *I*^2^ = 74.52%, Tau^2^ = 0.040) for Social Attribution Task-multiple choice, and 0.776 [0.398–0.931] (*k* = 2, *I*^2^ = 25.19%, Tau^2^ = 0.001) for Movie for the Assessment of Social Cognition. Internal consistency of Reading the Mind in the Eye Test (*k* = 31, *α* = 0.650 [0.604–0.692], *I*^2^ = 90.36%, Tau^2^ = 0.039), Hinting Task (*k* = 8, *α* = 0.554 [0.475–0.626], *I*^2^ = 69.53%, Tau^2^ = 0.017), the Short Stories Task (*k* = 4, *α* = 0.659 [0.522–0.765], *I*^2^ = 52.48%, Tau^2^ = 0.010), and Picture Sequencing Task (*k* = 2, *α* = 0.642 [− 0.843–0.998], *I*^2^ = 90.52%, Tau^2^ = 0.076) was not acceptable in NC. High levels of heterogeneity were exhibited in Reading the Mind in the Eye Test and Hinting Task for SZ, and Reading the Mind in the Eye Test, Hinting Task, The Awareness of Social Inference Test—Part three, Faux Pas Test, and Social Attribution Task-multiple choice for NC. Egger’s test and funnel plots revealed that only the internal consistency of Reading the Mind in the Eye Test in NC had a potential publication bias, yet the trim-and-fill method did not suggest any additional estimate (Table 4 and Supplementary Fig. 2). Comparisons of Cronbach’s alpha between SZ and NC were conducted for Hinting Task, Reading the Mind in the Eye Test, Social Attribution Task-multiple choice, and The Awareness of Social Inference Test—Part three. Only Hinting Task was found to exhibit a significant difference where the internal consistency of NC was significantly lower than that of SZ (*F*[1, 19] = 12.739, *p* = 0.002).

**Table 4:** Meta-analysis of Internal consistency in ToM tasks

Task	Population	*k*	*n*	Cronbach’s alpha (95% CI)	*Q*	*Q p* value	*I*^2^ Statistics, %	Tau^2^	*p* Egger
RMET	NC	31	7614	0.650 [0.604–0.692]	316.79	< 0.001	90.36	0.039	0.288
	SZ	3	435	0.694 [0.314–0.889]	6.25	0.044	79.55	0.032	**0.004**
	SZ-NC	3	339	**0.722 [0.666–0.771]**	0.24	0.887	0.00	0.000	0.154
HT	NC	8	1153	0.554 [0.465–0.634]	26.06	0.001	69.53	0.017	0.406
	SZ	13	1616	**0.713 [0.655–0.763]**	48.43	< 0.001	74.62	0.026	0.898
	SZ-NC	6	575	0.679 [0.633–0.720]	2.87	0.720	0.00	0.000	0.852
	ASD	2	131	**0.774 [0.042–0.989]**	1.49	0.223	32.70	0.012	N/A
TASIT-III	NC	5	631	**0.732 [0.600–0.827]**	26.11	< 0.001	78.82	0.034	0.867
	SZ	5	582	**0.798 [0.784–0.810]**	0.39	0.983	0.00	0.000	0.591
FPT	NC	5	936	**0.890 [0.734–0.957]**	110.10	< 0.001	96.03	0.148	0.897
TSST	NC	4	493	0.659 [0.522–0.765]	6.37	0.095	52.48	0.010	0.234
MASC	NC	2	566	**0.776 [0.398–0.931]**	1.34	0.248	25.19	0.001	N/A
	ASD-NC	2	87	**0.851 [0.669–0.938]**	0.10	0.746	0.00	0.010	N/A
SAT-MC	NC	3	265	**0.719 [0.323–0.906]**	6.70	0.035	74.52	0.040	0.801
	SZ	3	310	**0.817 [0.743–0.871]**	1.20	0.550	0.00	0.000	0.281
PST	NC	2	295	0.640 [–0.843–0.998]	10.55	0.001	90.52	0.076	N/A
	SZ	2	123	**0.839 [0.117–0.984]**	1.05	0.306	4.77	0.001	N/A
SST	NC	2	1334	0.666 [0.601–0.723]	0.10	0.746	0.00	0.000	N/A

Note: *ASD* indicates autism spectrum disorder; *SZ*, schizophrenia; *NC*, nonclinical population; *ASD-NC*, autism spectrum disorder and nonclinical population mixed together; *SZ-NC*, schizophrenia and nonclinical population mixed together; *RMET*, Reading the Mind in the Eye Test; *HT*, Hinting Task; *TASIT-III*, The Awareness of Social Inference Test – Part three; *FPT*, Faux Pas Test; *SAT-MC*, Social Attribution Task-multiple choice; *TSST*, The Short Story Task; *MASC*, Movie for the Assessment of Social Cognition. Bold indicates a satisfactory or above (> 0.70) reliability

Meta-regression analysis indicated that the standard deviations of task scores were a significant moderator of the Cronbach’s alpha of Reading the Mind in the Eye Test (*F*[1, 21] = 14.914, *p* < 0.001) and The Awareness of Social Inference Test—Part three in NC (*F*[1, 3] = 33.659, *p* = 0.010), where a higher standard deviation displayed a better internal consistency (Supplementary Table 3). For the participant-related moderators, mean age of participants had a positive moderating effect on the internal consistency of Hinting Task (*F*[1, 6] = 11.004, *p* = 0.016) and The Awareness of Social Inference Test—Part three (*F*[1, 3] = 46.960, *p* = 0.006) in NC. Additionally, years of education were also found to have a negative association with the internal consistency of Hinting Task in SZ (*F*[1, 7] = 14.740, *p* = 0.006).

### Test–Retest Reliability

The test–retest reliability of Movie for the Assessment of Social Cognition was 0.85 in SZ-NC (*k* = 1), whereas test–retest reliability of Faux Pas Test was 0.76 [0.61–0.86] (*k* = 1) in SZ (Table 3). For the SZ population, the test–retest reliability of Hinting Task (*k* = 5, *r* = 0.652 [0.587–0.709], *I*^2^ = 28.85%, Tau^2^ = 0.003), The Awareness of Social Inference Test—Part three (*k* = 5, *r* = 0.621 [0.454–0.746], *I*^2^ = 53.78%, Tau^2^ = 0.013), Social Attribution Task-multiple choice (*k* = 2, *r* = 0.636 [− 0.776–0.988], *I*^2^ = 56.08%, Tau^2^ = 0.025) and Picture Sequencing Task (*k* = 2, *r* = 0.586 [− 0.197–0.913], *I*^2^ = 0.00%, Tau^2^ = 0.000) was not acceptable (Table 5). Test–retest reliability of Reading the Mind in the Eye Test was acceptable in both SZ (*k* = 4, *r* = 0.731 [0.626–0.810], *I*^2^ = 73.16%, Tau^2^ = 0.026) and NC (*r*: *k* = 5, *r* = 0.713 [0.672–0.750], *I*^2^ = 2.77%, Tau^2^ = 0.001; ICC: *k* = 7, ICC = 0.762 [0.676–0.828], *I*^2^ = 92.98%, Tau^2^ = 0.054). While test–retest reliability was mainly analyzed using Pearson’s correlation (*r*) due to availability of data, and only Reading the Mind in the Eye Test in NC had enough studies for meta-analysis of intraclass correlation (ICC). For the NC population, test–retest reliability of The Awareness of Social Inference Test—Part three (*k* = 3, *r* = 0.624 [0.423–0.767], *I*^2^ = 79.94%, Tau^2^ = 0.048), Hinting Task (*k* = 4, *r* = 0.528 [0.467–0.584], *I*^2^ = 0.00%, Tau^2^ = 0.000), and Social Attribution Task-multiple choice (*k* = 1, *r* = 0.554) was poor. Only Movie for the Assessment of Social Cognition was evaluated with the test–retest reliability for ASD which had a correlation coefficient of 0.92 (*k* = 1). Heterogeneity of test–retest reliability was high in Reading the Mind in the Eye Test for SZ and NC, and in The Awareness of Social Inference Test—Part three for NC, but moderate in The Awareness of Social Inference Test—Part three and Social Attribution Task-multiple choice for SZ. Egger’s test and funnel plots did not indicate any potential publication bias (Table 5 and Supplementary Fig. 3). Similar to internal consistency, NC had significantly lower test–retest reliability compared with SZ in performing Hinting Task (*F*[1, 7] = 8.615, *p* = 0.022), but not Reading the Mind in the Eye Test and The Awareness of Social Inference Test—Part three.

**Table 5:** Meta-analysis of test–retest reliability in ToM tasks

Task	Population	*k*	*n*	Test–retest reliability (95% CI)	*Q*	*Q p* value	*I*^2^ statistics, %	Tau^2^	*p* Egger
RMET	NC	5	809	***r*: 0.713 [0.634–0.778]**	6.928	0.140	2.77	0.001	0.188
		7	1838	**ICC: 0.762 [0.652–0.841]**	93.73	< 0.001	92.98	0.054	0.562
	SZ	4	483	***r*: 0.731 [0.537–0.851]**	9.45	0.024	73.16	0.027	0.106
HT	NC	4	596	*r*: 0.528 [0.404–0.633]	4.40	0.221	0.00	0.000	0.535
	SZ	5	880	*r*: 0.652 [0.587–0.709]	4.80	0.309	28.85	0.003	0.216
TASIT-III	NC	3	296	*r*: 0.624 [0.081–0.881]	7.20	0.027	79.94	0.048	0.169
	SZ	5	538	*r*: 0.621 [0.454–0.746]	8.78	0.067	53.78	0.013	0.742
SAT-MC	SZ	2	250	*r*: 0.636 [− 0.776–0.988]	2.28	0.131	56.08	0.025	N/A
PST	SZ	2	95	*r*: 0.586 [− 0.197–0.913]	0.42	0.517	0.00	0.000	N/A

Note: *SZ*, schizophrenia; *NC*, nonclinical population; *RMET*, Reading the Mind in the Eye Test; *HT*, Hinting Task; *TASIT-III*, The Awareness of Social Inference Test—Part three; *FPT*, Faux Pas Test; *SAT-MC*, Social Attribution Task-multiple choice; *TSST*, The Short Story Task; *MASC*, Movie for the Assessment of Social Cognition; *r*, Pearson’s correlation; *ICC*, intraclass correlation. Bold indicates a satisfactory or above (> 0.70) reliability

Meta-regression analysis did not indicate any significant moderating effects of participant-related characteristics, mean and standard deviation of task scores, test–retest intervals, and other study characteristics (Supplementary Table 4).

### Inter-rater reliability

There were only twelve studies examining inter-rater reliability of eleven ToM tasks, whereas only the Faux Pas Test in NC had two studies (*k* = 2, ICC = 0.893 [0.316–0.990]; *I*^2^ = 63.91%). Studies examining the inter-rater reliability of Hinting Task, Adult-Theory of Mind, Faux Pas Test, and The Combined Stories Test involved the SZ population, and only studies with Movie for the Assessment of Social Cognition and the Short Stories Task involved the ASD population. These ToM tasks generally exhibited an acceptable inter-rater reliability across populations (Table 1).

## Discussion

The current study systematically evaluated the psychometric reliability of ToM tasks in schizophrenia (SZ), autism spectrum disorders (ASD), and nonclinical populations (NC) with 90 studies and 27 distinct ToM tasks being included. Our findings suggested that the psychometric reliability of ToM tasks varies substantially in clinical and nonclinical populations. In general, all ToM tasks were found to have acceptable internal consistency in SZ and ASD. However, only The Awareness of Social Inference Test—Part three, Faux Pas Test, Social Attribution Task-multiple choice, and Movie for the Assessment of Social Cognition were found to have adequate internal consistency in NC. Additionally, only Faux Pas Test and Movie for the Assessment of Social Cognition demonstrated sufficient internal consistency and test–retest reliability across populations, albeit with limited number of studies. Also, Hinting Task had satisfactory internal consistency in SZ and ASD but not in NC, and its test–retest reliability was poor in SZ and NC, respectively. Available evidence suggested that ToM tasks that involved manual rating generally exhibited good inter-rater reliability. However, studies were limited. Meta-regression analysis revealed that the standard deviation of task scores was a significant moderator on the internal consistency of Reading the Mind in the Eye Test and The Awareness of Social Inference Test—Part three in NC. Age also significantly influenced the internal consistency of Hinting Task and The Awareness of Social Inference Test—Part three in NC, while years of education significantly impacted the internal consistency of Hinting Task in SZ, suggesting that demographics should be taken into account for interpretations of these specific tasks. No ToM task exhibited excellent psychometric reliability across populations. This comprehensive review of the psychometric reliability of ToM tasks in specific populations and mixed-populations offered practical implications for the application and interpretation of these tasks in both research and clinical settings. It also highlighted the need for additional research to generate more reliable and robust data, particularly in the psychometric evaluations of ToM tasks in ASD adults which only had ten studies in total with two focusing on test–retest reliability.

Findings herein also suggested that ToM tasks have different psychometric properties between clinical and nonclinical populations, with the clinical population tend to demonstrate satisfactory psychometric reliability but not the nonclinical populations (Gourlay et al., 2020; Morrison et al., 2019; Pinkham et al., 2018). The different psychometric properties of the same task across different populations might limit the comparability of the task results between different populations and also shed doubts on the suitability of the usage of the tasks. Specifically, we found that the nonclinical population presented significantly lower internal consistency and test–retest reliability than SZ in the Hinting Task. Reading the Mind in the Eye Test also displayed marginal internal consistency in NC. However, Reading the Mind in the Eye Test and Hinting Task have been used frequently for comparisons between clinical and nonclinical populations in existing studies. A study by Klein et al. (2022) found poor psychometric properties in various ToM tasks among nonclinical undergraduates, including a prominent ceiling effect in Hinting Task for one-fifth of the participants. The psychometric reliability of Hinting Task did not significantly improve despite efforts to refine the scoring method (Klein et al., 2020). A review by Yeung et al. (2024) also indicated about half of the ToM tasks exhibited a ceiling effect for at least one subscale in neurotypical populations. The presence of ceiling effects in ToM tasks particularly in neurotypical populations would significantly reduce the variance in observed test scores, consequently leading to a lower internal consistency and test–retest reliability as suggested by classical test theory (DeVellis, 2006). Our meta-regression results also indicated positive correlations between internal consistency with the standard deviation of task scores. These findings raised an important consideration for the application of existing ToM tasks in the nonclinical population for different research contexts, including studies of developmental trajectory, individual differences, and subclinical symptoms in the general populations.

While poor and differential psychometric reliability among populations might indicate a potential issue of measurement invariance where ToM tasks may not be measuring the same construct in the same way across different populations (Widaman & Reise, 1997), the group-specific response style or variance may also contribute to these observed differences. It is possible that these ToM tasks were primarily designed for ASD and SZ populations who are likely to have prominent social cognition deficits. Thus, the tasks present measurement sensitivity in detecting “ToM impairments,” rather than capturing the general variation in “ToM ability” which, similar to neurocognitive functions, should be diverse in the general populations (Conway et al., 2019; Fu et al., 2023; Gernsbacher & Yergeau, 2019; Hayward & Homer, 2017; Holt et al., 2022; Marocchini, 2023; Yeung et al., 2024). Individual differences in ToM ability might reflect how easily and fluently adults attribute mental states to others (Hughes & Devine, 2015), or how individuals build internal representations of others’ mental states based on a multidimensional “Mind-space” framework (Conway et al., 2019). The inability to capture these variations in the neurotypical population with the existing ToM tasks would lead to ceiling effects, thus compromising the psychometric reliability of these tasks. Despite the importance to capturing individual differences of ToM ability, significant gaps persist in the development of conceptual framework and measurement tools in this area (Fu et al., 2023; Osterhaus & Bosacki, 2022; Warnell & Redcay, 2019; Yeung et al., 2024). Effectively examining the broad spectrum of ToM ability could also be valuable to understand the psychological and neurobiological basis of social interactions, and establish benchmarks for abnormal deviations. Therefore, it is crucial to develop ToM tasks that can sensitively capture the variability in ToM abilities as well as impairments across both clinical and nonclinical populations, complemented by the continuous conceptual refinement of the ToM.

Our study found temporal consistency of Reading the Mind in the Eye Test and Movie for the Assessment of Social Cognition was satisfactory across populations, whereas Faux Pas Test also showed sufficient test–retest reliability specifically in the SZ population. Thus, these tasks are suitable for longitudinal and interventional studies with their respective populations. Results also found the inadequate test–retest reliability in Hinting Task, The Awareness of Social Inference Test—Part three, Social Attribution Task-multiple choice, and Picture Sequencing Task in SZ and NC, indicating that the observed scores were significantly impacted by potential measurement and random error. While The Awareness of Social Inference Test—Part three and Social Attribution Task-multiple choice displayed adequate to good internal consistency across populations, the unsatisfactory test–retest reliability cast doubts on the ability of these tasks to detect genuine variations of ToM over time or changes after interventions. Notably, the variability in test–retest reliability of Hinting Task, Reading the Mind in the Eye Test, and The Awareness of Social Inference Test—Part three among studies in both SZ and NC was not significantly influenced by the spacing between measurement intervals. The poor test–retest reliability of Hinting Task could be particularly affected by the practice effect due to its experimental design that offers hints on second attempts (Klein et al., 2020; Ludwig et al., 2017; Pinkham et al., 2016, 2018). Participants may remember and utilize hints from their first attempts for their subsequent attempts and thus artificially boost their performance and compromise the task’s reliability for repeated measurements. Additionally, the use of nonequivalent alternates forms in Social Attribution Task-multiple choice and The Awareness of Social Inference Test—Part three could be the cause of unsatisfactory test–retest reliability (Davidson et al., 2018; Johannesen et al., 2018; Ludwig et al., 2017; Pinkham et al., 2016, 2018). Furthermore, only two studies have examined the test–retest reliability of ToM tasks in ASD adults, and only involved Movie for the Assessment of Social Cognition and Adult-Theory of Mind (Table 1). Therefore, further research efforts on the psychometric properties specifically on the test–retest reliability of ToM tasks in the ASD population would be important.

The present systematic review and meta-analysis also pointed out that no single ToM task to date has exhibited excellent psychometric reliability across populations with sufficient studies. According to Nunnally and Bernstein (1994), the preferred reliability for a clinical test ranges from 0.9 to 0.95, although lower values may still be acceptable for research purposes. Precision in ToM assessments as well as other similar socioemotional constructs are crucial in both research and clinical applications. Meanwhile, we recommend the use of the Faux Pas Test and Movie for the Assessment of Social Cognition for both clinical and research purposes due to their generally satisfactory psychometric reliability based on the existing evidence. However, it is important to note that the volume of psychometric research on both tasks remains limited, particularly for the Faux Pas Test among adults with ASD. Therefore, more comprehensive validation and further studies are essential to confirm their efficacy and reliability across different populations and contexts. Moreover, preliminary results of some new ToM tasks show promising psychometric properties, such as the short version of Faux Pas Test and the video-based Assessment of ToM for people with Schizophrenia (Yeh et al., 2023) (Table 1), warranting further research and validation studies to establish their utility.

Reliability and validity of ToM tasks are inter-related with reliability being the prerequisite of validity. The diverse operationalizations of included ToM tasks, which vary in modalities and formats, raised concerns about the validity of direct comparisons between tasks and across studies. A recent review by Yeung et al. (2024) pointed out that ToM tasks presented poor and inconsistent inter-correlations, suggesting the presence of jingle fallacies where most of the ToM tasks were actually measuring different constructs (Olderbak & Wilhelm, 2020). These findings suggested that ToM should be regarded as a multi-dimensional, instead of a single construct, yet there was still a lack of consensus and robust empirical support on its conceptual frameworks (Osterhaus & Bosacki, 2022; Warnell & Redcay, 2019; Yeung et al., 2024). Studies also indicated unresolved issues on convergent and discriminant validity between ToM tasks with other socio-emotional constructs, such as empathy, agreeableness, and emotion perception (Bainbridge et al., 2022; Kittel et al., 2022; Olderbak & Wilhelm, 2017; Pavlova & Sokolov, 2022). These fundamental differences among ToM tasks could significantly influence their psychometric properties, including internal consistency and test–retest reliability. Theoretically, a ToM task characterized by complex formats as well as a broad and loosely defined construct would be associated with poorer reliability, whereas a simpler task with precise and well-defined construct is more likely to have better reliability. Compared to the relatively psychometrically well-established neuropsychological tests for general cognitive functions (Strauss et al., 2006), more conceptual development in the field of social cognition and ToM in parallel with the development of assessment tools with satisfactory psychometric properties would be crucial (Fu et al., 2023; Yeung et al., 2024). Besides, ToM has been conceptualized as a complex construct with dimensions that can be captured by other assessment approaches beyond mere accuracy, including reaction time, eye-tracking parameters, and self-report measures (Chan et al., 2022; Olderbak & Wilhelm, 2020). While our focus on the psychometric reliability of accuracy-based behavioral tasks is essential, other approaches assessing ToM can enrich our understanding of the nuances of ToM by capturing different dimensions of the construct. The psychometric properties of these approaches should also be assessed in future studies to ensure their reliability and validity in diverse settings and populations.

### Limitations

As our focus of this review was on the reliability of ToM tasks, we did not critically examine the construct and ecological validity of these ToM tasks. While we preliminarily assessed the construct validity of tasks employing the practical framework proposed by Quesque and Rossetti (2020) to supplement our evaluation of psychometric reliability (Table 1), we acknowledge that the suggested mentalizing and nonmerging criteria might present limitations. Particularly, the self-other differentiation of the nonmerging criteria has been regarded as an ambiguous and debatable concept. It is nearly impossible to distance oneself and understand others without incorporating one’s own experiences, which are fundamental to perspective taking (Conway et al., 2019). It also overlooks other critical constructs relevant to a comprehensive understanding of ToM, such as affective ToM and social pragmatics. Also, it is important to emphasize again that Cronbach’s alpha has significant limitations and is not considered a sufficient psychometric standard for clinical and research settings on its own (Sijtsma, 2009). Direct tests of measurement invariance, such as multi-group confirmatory factor analysis, are recommended as a more effective method for understanding the underlying structures of measurement construct across populations and provide evidence on this issue.

Moreover, the number of psychometric evaluation studies reporting psychometric reliability, particularly test–retest reliability, was scarce for some tasks among the ASD population. The study sizes for meta-regression analyses were limited and should be cautiously interpreted. Some commonly used tasks, such as Picture Sequencing Task and Faux Pas Test, did not have any test–retest reliability evaluation in ASD and nonclinical populations. Results based on few studies (*k* = 1–3) should be interpreted cautiously due to their potential instability and limited generalizability. Hence, more psychometric evaluations dedicated to different tasks and populations should be conducted, and future studies were encouraged to document psychometric reliability in a standardized format albeit not being the main focus of the paper. Our study also included studies with test–retest intervals up to 365 days, which might be better interpreted as measuring the long-term stability of ToM rather than immediate test–retest reliability. However, there is no established standard for the optimal interval to assess test–retest reliability, particularly for psychological constructs. To address the potential impact of these varying intervals, we have conducted meta-regression analyses using test–retest intervals as moderator with no significant moderating effect found.

Additionally, while this review included only peer-reviewed journal articles to ensure the consistency and verifiability of the data analysis approaches, we acknowledge the possibility of publication bias by excluding research findings from grey literature, such as unpublished data and dissertations. Future studies should consider incorporating unpublished works to provide a more comprehensive overview and mitigate the possibility of publication bias. The current review exclusively focused on the psychometric reliability in SZ, ASD, and nonclinical populations. Hence, the results might not be generalized in other clinical populations. Only English literature was included, and this would have limited the generalizability of the review and also limited the detection of possible cultural variation on psychometric variation of ToM tasks.

### Conclusion

To our best knowledge, this is the first quantitative analysis to evaluate the internal consistency and test–retest reliability of ToM tasks in schizophrenia (SZ), autism spectrum disorder (ASD), and nonclinical populations (NC). Overall, Faux Pas Test and Movie for the Assessment of Social Cognition showed satisfactory internal consistency and test–retest reliability across populations albeit limited evidence. Reading the Mind in the Eye Test generally displayed acceptable internal consistency and test–retest reliability across populations, though it showed only marginal internal consistency in nonclinical samples. The Awareness of Social Inference Test—Part three and Social Attribution Task-multiple choice also had an adequate internal consistency but poor test–retest reliability. Most ToM tasks had acceptable internal consistency in the SZ and ASD population but not for NC. Psychometric studies of ToM tasks in the ASD adult population are generally limited, particularly on test–retest reliability. Results on the overall psychometric reliability of the existing ToM tasks in NC, ASD, and SZ may provide guidance on the selection of tasks in clinical and research settings. Varying psychometric reliabilities across populations also indicated potential presence of measurement invariance and group-specific variance. Developing ToM tasks capable of effectively capturing the spectrum of ToM abilities as well as the development in operationalization and conceptualization of ToM are needed.

## Supplementary Information

Below is the link to the electronic supplementary material.

Supplementary file1 (DOCX 318 KB)

## Data Availability

The data file and analysis code for this study are openly available in the Open Science Framework (OSF) repository at https://osf.io/sj746/?view_only=68c65ff7db8541edb3c952699e4e8d7f.
