# Supplementary material for: Reliability of Theory of Mind Tasks in Schizophrenia, ASD, and Nonclinical Populations: A Systematic Review and Reliability Generalization Meta-analysis
Source: Neuropsychol Rev. 2024 Oct 8;35(3):552–68. doi: 10.1007/s11065-024-09652-4 (PMC12602658; doi:10.1007/s11065-024-09652-4)
Supplement: Supplementary file 1 — Supplementary file1 (DOCX 318 KB) [file 11065_2024_9652_MOESM1_ESM.docx]

Supplementary information for

Reliability of Theory of Mind Tasks in Schizophrenia, ASD, and Nonclinical Populations: a systematic review and reliability generalization meta-analysis

[**Supplementary Table 1**. Demographics, sample size, and reported reliability estimates of other included ToM tasks. 2](#_Toc166172292)

[**Supplementary Table 2.** Overview of included studies. 4](#_Toc166172293)

[a) Studies involved NC only 4](#_Toc166172294)

[b) Studies involved SZ 6](#_Toc166172295)

[c) Studies involved ASD 8](#_Toc166172296)

[**Supplementary Table 3.** Meta-regression analyses on internal reliability (Cronbach’s alpha). 10](#_Toc166172297)

[**Supplementary Table 4.** Meta-regression analyses on test-retest reliability (Pearson’s r, if not specified). 12](#_Toc166172298)

[**Supplementary Table 5.** Quality assessment of the included studies**.** 14](#_Toc166172299)

[**Supplementary Figure 1.** A summary of the risk-of-bias evaluation. 19](#_Toc166172300)

[**Supplementary Figure 2.** Funnel plots of the meta-analyses of internal consistency. 20](#_Toc166172301)

[**Supplementary Figure 3.** Funnel plots of the meta-analyses of test-retest reliability. 24](#_Toc166172302)

[References of included studies 27](#_Toc166172303)

[**Supplementary Material 1.** PRISMA 2020 Checklist. 35](#_Toc166172304)

[**Supplementary Material 2.** REGEMA Checklist. 38](#_Toc166172305)

## **Supplementary Table 1**. Demographics, sample size, and reported reliability estimates of other included ToM tasks.

| **Task** | **Population** | **n** | **N in total**  **(N of HC)** | **Age, mean** | **Gender (male %)** | **Education, mean** | **IC (N)** | **TRR (N)** | **IRR (N)** |
| --- | --- | --- | --- | --- | --- | --- | --- | --- | --- |
| A-ToM | ASD | 1 | 163 | 27 | 69.3 | NR |  | 0.82 (1; Kappa) |  |
| AToMs | SZ | 1 | 59 | 44.78 | 54.2 | 12.39 | 0.85 (1) | 0.901 (1) | 0.997 (1) |
| AT-MC | HC | 2 | 1831 | 21.99 | 42.0 | NR | 0.62 [-0.98-0.99] (2) |  |  |
| AT-verbal | HC | 1 | 1546 | 16 | 39.0 | NR | 0.87 (1) | 0.54 (1) |  |
|  | ASD-HC | 1 | 97 (58) | 45.40, 43.62 | 75.9, 56.5 | NR |  | 0.72 (1) |  |
| BAT | SZ-HC | 1 | 60 (30) | 45.5, 43.4 | 80.0%, 46.7% | NR | 0.85 (alpha),  0.89 (omega) |  |  |
| COST | HC | 1 | 42 | 25.1 | 35.7 | 16.9 |  | 0.84 (1) | 0.75 (1; Kappa) |
|  | SZ-HC | 1 | 62 (31) | 24.9, 25.2 | 83.9, 83.9 | NR | 0.81 (1) |  | 0.98 (1) |
| CST 14-item | HC | 1 | 20.09 | 46.7 | 13.46 | NR | 0.70 |  |  |
| CST 34-item | SZ | 2 | 191 | 41.2 | 68.0 | 12.45 | 0.74 [0.66-0.83] (1) | 0.71 (1) |  |
| CST 48-item | SZ-HC | 1 | 105 (19) | 29.0, 30.11 | 47.4, 36.0 | NR | 0.843 (1) | 0.572 (1) |  |
| FPT-short | HC | 2 | 202 | 29.51 | 39.0 | NR | 0.95 [0.90-0.97] (2) | 0.97 | 0.82 (Kappa) |
|  | SZ | 2 | 84 | 40.14 | 56 | NR | 0.7286 [-0.99-0.99] (2) | 0.78 | 0.87 (Kappa) |
| PST-modified | ASD-HC | 1 | 97 (58) | 45.40, 43.62 | 75.9, 56.5 | NR | 0.71-0.78 (1) |  |  |
| SKT | HC | 1 | 42 | 25.1 | 35.7 | 16.9 |  | 0.77 (1) | 0.99 (1; Kappa) |
| SST | HC | 2 | 1334 | 32.73 | 48.7 | NR | 0.67 (2) |  |  |
|  | ASD | 1 | 229 | 33.61 | 44.5 | NR | 0.72 [0.67-0.77] (1) |  |  |
| SSFT | ASD-HC | 2 | 117 (78) | 40.47, 39.30 | 83.9, 69.3 | NR | Mental state: 0.42 (1)  Extended mental state: 0.62 (1) |  |  |
| Strange stories | SZ | 1 | 53 | 42.3 | 77.4 | NR |  | 0.50 [0.24-0.70] (1) |  |
| Strange stories-revised | HC | 1 | 100 | 47.7 | 50.0 | 15.34 | 0.63 (1) |  | 0.929 [0.892-0.953] (1) |
| ToM-HCAT (Aykan & Nalçacı, 2018) | HC | 1 | 103 | 19.68 | 44.7 | NR | 0.94 (1) |  |  |
| VAMA | HC | 1 | 65 | 25.98 | 38.5 | 14.19 | 0.69-0.84 (1) | 0.93 [0.85-0.96] (1; ICC) |  |
| Yoni Task | HC | 1 | 235 | 41.46 | 46.0 | 15.29 | 0.90 (1) |  |  |
| V-SIR | SZ | 1 | 143 | 31.4 | 77.6 | 12.6 | 0.74 [0.68] (1) |  |  |

Note. ASD indicates autism-spectrum disorder; SZ, schizophrenia; NC, nonclinical population; ASD-NC, autism-spectrum disorder and nonclinical population mixed together; SZ-NC, schizophrenia and nonclinical population mixed together; TRR, test-retest reliability; IC, internal consistency; IRR, inter-rater reliability; ICC, intraclass correlation; NR, not reported; A-ToM, Adult-Theory of Mind; AToMs, Assessment of ToM for people with Schizophrenia; AT-MC, Animated Triangles Task-MC; AT-verbal, Animated Triangles Task-verbal; BAT, Battery for the Assessment of ToM; COST, The Combined Stories Test; CST, Comic Strip Task; FPT, Faux Pas Test; PST, Picture Sequencing Task; SKT, Social Knowledge Test; SST, Social shape task; SSFT, Strange Stories film task; ToM-HCAT, The Humor Comprehension and Appreciation Test; VAMA, The Virtual Assessment of Mentalising Ability; V-SIR, Versailles-Situational Intention Reading. n indicates the number of studies, and N indicates the sample size.

## **Supplementary Table 2.** Overview of included studies.

### a) Studies involved NC only

| Author, Publication Year | Country of study | Sample Size | Populations | Age, mean | Gender (Male %) | Assessment conducted | Test-retest reliability | Internal Consistency | Inter-rater reliability |
| --- | --- | --- | --- | --- | --- | --- | --- | --- | --- |
| Ali & Chamorro-Premuzic, 2010 | UK | 112 | NC | 20.74 | 17.9% | RMET |  | Y |  |
| Andersen et al., 2022 | Denmark | 1546 | NC | 16 | 45.0% | AT-MC, -verbal |  | Y | Y |
| Aykan & Nalçacı, 2018 | Turkey | 103 | NC | 19.68 | 44.7% | ToM-HCAT |  | Y |  |
| Bedwell et al., 2014 | US | 686 | NC | 21.22 | 33.0% | RMET |  | Y |  |
| Brown et al., 2019 | US | 505 | NC | 35.76 | 56.0% | SST |  | Y |  |
| Canty et al., 2015 | Australia | 65 | NC | 25.98 | 38.5% | VAMA | Y (Interval days = 28; N = 30) | Y |  |
| Dehning et al., 2012 | Ethiopia | 237 | NC | 19.3, 24 | 87.3% | RMET |  | Y |  |
| Dodell-Feder et al., 2013 | US | 74 | NC | 27.8 | 36.49% | TSST |  | Y | Y |
| Faísca et al., 2016 | Portugue | 200 | NC | 33 | 37.5% | FPT, PST |  | Y |  |
| Ferguson et al., 2010 | UK | 99 | NC | 34.08 | 29.0% | FPT, RMET |  | Y |  |
| Fernández-Abascal et al., 2013 | Spain | 358 | NC | 34.23 | 20.9% | RMET | Y (Interval days = 365) |  |  |
| Fossati et al., 2018 | Italy | 373, 193 | NC | 17.13, 32.77 | 36.2%, 40.7% | MASC, RMET |  | Y |  |
| Giordano et al., 2019 | Mexico | 118 | HC | 23.03 | 38.14% | RMET, TSST |  | Y |  |
| Girli et al., 2014 | Turkey | 268 | NC |  | 40.0% | RMET |  | Y |  |
| Gong et al., 2014 | China | 329 | NC | 22.3 | 38.6% | RMET |  | Y |  |
| Gourlay et al., 2020 | Canada | 100 | NC | 47.7 | 50.0% | Strange stories-Revised |  | Y | Y |
| Harkness et al., 2010 | Canada | 93 | NC | 18.9 | 18.3% | RMET |  | Y |  |
| Higgins et al., 2023 | US | 1181 | NC | 47.7 | 44.8% | RMET |  | Y |  |
| Isernia et al., 2023 | Italy | 235 | NC | 41.46 | 46.0% | Yoni |  | Y |  |
| Jankowiak-Suida et al., 2016 | Poland | 325 | NC | 27.85 | 50.5% | RMET | Y (Interval days = 28) | Y |  |
| Johannesen et al., 2013 | US | 51 | NC | 19.27 | 39.2$% | SAT-MC |  | Y |  |
| Khorashad et al., 2015 | Iran | 545 | NC | 25.8 | 48.3% | RMET | Y (Interval days = 365) | Y |  |
| Klein et al., 2022 | US | 265 | NC | 21.19 | 53.2% | HT, RMET, TASIT-III |  | Y |  |
| Koo et al., 2020 | Korea | 196 | NC | 23.02 | 17.9% | RMET | Y (Interval days = 13.96; N = 25) | Y |  |
| Le Donne et al., 2023 | Italy | 261 | NC | 20.09 | 46.70% | CST 14-item |  | Y |  |
| Lee et al., 2020 | Korea | 200 | NC | 23.07 | 69.3% | RMET | Y (Interval days = 28; N = 27) |  |  |
| Livingston et al., 2021 | UK | 285 | NC | 27.98 | 20.0% | AT-MC |  | Y |  |
| Mar et al., 2006 | Canada | 94 | NC | 22.3 | 58.3% | RMET |  | Y |  |
| Megías-Robles et al., 2020 | Spain | 874 | NC | 22.44 | 58.3% | RMET |  | Y |  |
| Melchers et al., 2015 | Germany | 108 | NC | 22.27 | 87.3% | RMET |  | Y |  |
| Navarro, 2022 | US | 208 | HC | 39.89 | 44.23% | RMET, TSST |  | Y |  |
| Pfaltz et al., 2013 | Germany | 155 | NC | 31.2 | 36.2% | RMET | Y (Interval days = 21; N = 40) |  |  |
| Preller et al., 2015 | Switzerland | 68 | NC | 29.81 | 76.4% | RMET |  | Y |  |
| Prevost et al., 2014 | France | 97, 109 | NC | 31.3, 31.6 | 56.1%, 46.0% | RMET | Y (Interval days = 7; N = 30) | Y |  |
| Şandor & İşcen, 2023 | Turkey | 416 | NC | 32.52 | 65.7% | FPT |  | Y | Y |
| Smeets et al., 2009 | Germany | 64 | NC | 25.89 | 53.2% | RMET |  | Y |  |
| Söderstrand & Almkvist, 2012 | Sweden | 68 | NC | 34.8 | 53.2% | FPT, RMET |  | Y | Y |
| Thibaudeau et al., 2018 | Canada | 42 | NC | 25.1 | 17.9% | COST, SKT | Y (Interval days = 27.6) |  | Y |
| Thoma et al., 2014 | Germany | 20 | NC | 45.65 | 45.0% | RMET |  | Y |  |
| Turner & Vallée-Tourangeau, 2020 | UK | 93 | HC | 24.73 | 19.35% | TSST |  | Y | Y |
| Van Doesum et al., 2013 | Netherlands | 225 | NC | 34.05 | 44.7% | RMET |  | Y |  |
| Vellante et al., 2013 | Italy | 200 | NC | 24.1 | 65.8% | RMET | Y (Interval days = 30) | Y |  |
| Voracek & Dressler, 2005 | Austria | 423 | NC | 29.7 | 60.6% | RMET |  | Y |  |
| Watanabe et al., 2021 | Brazil | 153 | NC | 38.8 | 80.56%, 80% | FPT |  | Y |  |
| Yildirim et al., 2011 | Turkey | 117 | NC | 33.33 | 56.0% | RMET | Y (Interval days = 14; N = 70) |  |  |

Note. ASD indicates autism-spectrum disorder; SZ, schizophrenia; NC, nonclinical population; ASD-NC, autism-spectrum disorder and nonclinical population mixed together; SZ-NC, schizophrenia and nonclinical population mixed together; Y, reported; RMET, Reading the Mind in the Eye Test; HT, Hinting Task; TASIT-III, The Awareness of Social Inference Test - Part three; FPT, Faux Pas Test; SAT-MC, Social Attribution Task-multiple choice; TSST, The Short Story Task; MASC, Movie for the Assessment of Social Cognition; COST, The Combined Stories Test; CST, Comic Strip Task; PST, Picture Sequencing Task; SKT, Social Knowledge Test; SST, Social shape task; ToM-HCAT, The Humor Comprehension and Appreciation Test; VAMA, The Virtual Assessment of Mentalising Ability.

### b) Studies involved SZ

| Author, Publication Year | Country of study | Sample Size | Populations | Age, mean | Gender (Male %) | Assessment conducted | Test-retest reliability | Internal Consistency | Inter-rater reliability |
| --- | --- | --- | --- | --- | --- | --- | --- | --- | --- |
| Achim et al., 2012 | Canada | 31, 31 | SZ, NC | 24.9, 25.2 | 83.9% | COST |  | Y | Y |
| Bechi et al., 2012 | Italy | 76 | SZ | 38.38 | 65.8% | PST |  | Y |  |
| Bell et al., 2010 | US | 66, 85 | SZ, NC | 42.73 | 60.6% | SAT-MC |  | Y |  |
| Bozikas et al., 2011 | Greece | 36, 30 | SZ, NC | 36.72, 37.40 | 80.56%, 80% | HT |  | Y |  |
| Brunet-Gouet et al., 2021 | France | 143 | SZ | 31.4 | 77.6% | CST 34-item, V-SIR |  | Y |  |
| Charernboon & Lerthattasilp, 2017 | Thailand | 30,40 | SZ, NC | 37.9, 35.5 | 36.7%, 7.5% | RMET | Y (Interval days = 14) | Y |  |
| Chen et al., 2017 | Taiwan | 53 | SZ | 42.3 | 77.4% | FPT, RMET, Strange stories | Y (Interval days = 28) |  |  |
| Cruz et al., 2022 | Brazil | 104, 89 | SZ, NC | 41.99, 40.23 | 56.6%, 55.1% | HT |  | Y |  |
| Davidson et al., 2018 | US | 48 | SZ | 51 | 58.3% | CST 34-item, HT, PST, RMET, TASIT-III | Y (Interval days = 33.19) |  |  |
| Fekete et al., 2022 | Hungary | 47 | SZ | 41.19 | 48.9% | PST | Y (Interval days = 120) | Y |  |
| Fernández-Modamio et al., 2018 | Spain | 40, 50 | SZ, NC | 41.28, 37.02 | 52.5%, 36.0% | FPT-10 | Y (Interval days = 14) | Y | Y |
| Frøyhaug et al., 2019 | Norway | 30, 183 | SZ, HC | 27, 34 | 60.0%, 55.7% | HT |  | Y |  |
| Gil et al., 2012 | Spain | 40, 39 | SZ, NC | 42.38, 35.86 | 57.5%, 53.6% | HT | Y (Interval days = 15) | Y | Y |
| Han et al., 2020 | Korea | 158 | SZ | 39.5, 41.6 | 67.1% | HT |  | Y |  |
| Horan et al., 2012 | US | 55 | SZ | 22.3 | 76.4% | TASIT-III | Y (Interval days = 360) |  |  |
| Huang et al., 2023 | Taiwan | 41, 126 | SZ, NC | 47.61, 26.05 | 56.1%, 30.2% | MASC | Y (Interval days = 52.61; NC = 86) | Y |  |
| Johannesen et al., 2018 | US | 32 | SZ | 48.75 | 59.4% | SAT-MC | Y (Interval days = 15) | Y |  |
| Klein et al., 2020 | US | 397, 300 | SZ, NC | 41.9, 41.15 | 65.7%, 56.7% | HT | Y (Interval days = 15) | Y |  |
| Krawczyk et al., 2020 | Poland | 50, 50 | SZ, NC |  | 45.0% | HT |  | Y |  |
| Lee et al., 2018 | Korea, US | 60, 60 | SZ, NC | 33.79, 32 | 65.8%, 33.0% | HT, RMET, SAT-MC |  | Y |  |
| Lim et al., 2020 | Singapore | 116, 70 | SZ, NC | 38.29, 32.78 | 56.0%, 44.5% | HT, TASIT-III | Y (Interval days = 28) | Y |  |
| Lima-Sánchez et al., 2019 | Mexico | 19, 86 | SZ, NC | 29 | 38.5% | CST 48-item | Y (Interval days = 60; N = 80) | Y |  |
| Ludwig et al., 2017 | US | 38, 38 | SZ, NC | 23.45, 23.77 | 77.4%, 56.6% | HT, RMET, TASIT-III | Y (Interval days = 28) | Y |  |
| Mallawaarachchi et al., 2019 | Australia | 132 | SZ | 20.5 | 58.3% | HT |  | Y |  |
| Negrão et al., 2016 | Brazil | 44, 152 | SZ, NC | 39, 22 | 48.9%, 29.0% | FPT-10 |  | Y |  |
| Ng et al., 2015 | US | 193 | SZ | 46.19 | 29.0% | HT |  | Y |  |
| Park, 2018 | Korea | 51, 45 | SZ, NC | 37.45,30.06 | 36.0% | HT |  | Y |  |
| Pinkham et al., 2016 | US | 179, 104 | SZ, NC | 42.11, 39.2 | 40.7%, 36.2% | HT, RMET, TASIT-III | Y (Interval days = 17.29) | Y |  |
| Pinkham et al., 2018 | US | 218, 154 | SZ, NC | 41.72, 41.95 | 57.5%, 40.0% | HT, RMET, TASIT-III, SAT-MC | Y (Interval days = 16.69) | Y |  |
| Roberts & Penn, 2009 | US | 31 | SZ | 36.8, 41.4 | 58.06% | HT, TASIT-III |  | Y |  |
| Sedgwick et al., 2021 | UK | 14 | SZ | 45.1 | 56.7% | HT |  | Y |  |
| Serra-Mayoral et al., 2021 | Spain | 30, 30 | SZ, NC | 45.5, 43.4 | 80%, 46.7% | BAT |  | Y |  |
| Vidarsdottir et al., 2019 | Iceland | 70 | SZ | 24.1 | 33.0% | HT |  | Y |  |
| Yeh et al., 2023 | Taiwan | 59 | SZ | 44.78 | 69.3% | AToMs | Y (Interval days = 35) | Y | Y |
| Zhu et al., 2007 | China | 40, 31 | SZ, NC | 30.2, 29.97 | 44.5% | FPT | Y (Interval days = 90) |  | Y |

Note. SZ, schizophrenia; NC, nonclinical population; SZ-NC, schizophrenia and nonclinical population mixed together; Y, reported; RMET, Reading the Mind in the Eye Test; HT, Hinting Task; TASIT-III, The Awareness of Social Inference Test - Part three; FPT, Faux Pas Test; FPT-10, Faux Pas Test short version; SAT-MC, Social Attribution Task-multiple choice; TSST, The Short Story Task; MASC, Movie for the Assessment of Social Cognition; AToMs, Assessment of ToM for people with Schizophrenia; AT-MC, Animated Triangles Task-MC; AT-verbal, Animated Triangles Task-verbal; BAT, Battery for the Assessment of ToM; COST, The Combined Stories Test; CST, Comic Strip Task; PST, Picture Sequencing Task; V-SIR, Versailles-Situational Intention Reading.

### c) Studies involved ASD

| Author, Publication Year | Country of study | Sample Size | Populations | Age, mean | Gender (Male %) | Assessment conducted | Test-retest reliability | Internal Consistency | Inter-rater reliability |
| --- | --- | --- | --- | --- | --- | --- | --- | --- | --- |
| Adler et al., 2010 | Israel | 16,21 | ASD, NC | 21.87, 22.90 | 93.78%, 95.24% | RMET |  | Y |  |
| Brewer et al., 2017 | US | 163 | ASD | 27 | 69.3% | A-ToM | Y (Interval days = 165.9; N = 40) |  | Y |
| Brown et al., 2023 | US | 229, 829 | ASD, NC | 33.61, 29.69 | 44.5%, 41.4% | SST |  | Y |  |
| Dziobek et al., 2006 | Germany | 19, 20 | ASD, NC | 41.6, 39.9 | 90.5%, 90.0% | MASC | Y (Interval days = 138; N = 5) | Y | Y |
| Jarvers et al., 2023 | Germany | 32,32 | ASD, HC | 30.34, 31.13 | 62.5%, 53.10% | TSST |  | Y | Y |
| Lahera et al., 2016 | Spain | 22, 26 | ASD, NC | 21.94, 22.92 | 86.4%, 65.4% | MASC | Y (Interval days = 123; N = 5) | Y |  |
| Morrison et al., 2019 | US | 103, 95 | ASD, NC | 24.28, 24.17 | 90.5%, 88.4% | HT, PST, TASIT-III, RMET |  | Y |  |
| Murray et al., 2017 | UK | 20,20 | ASD, NC | 30.60,30.65 | 100%,95% | SSFT |  | Y |  |
| Pallathra et al., 2018 | US | 28 | ASD | 26 | 52.5% | HT |  | Y |  |
| Zıvralı Yarar et al., 2021 | UK | 29, 29, 20,19 | ASD **Y,** ASD O, NC Y, NC O | 29.48, 61.32, 29.40, 57.83 | 75.9%, 75.9%, 55.0%, 57.9% | ATT-verbal, PST-modified, SSFT |  | Y | Y |

Note. ASD indicates autism-spectrum disorder; NC, nonclinical population; ASD-NC, autism-spectrum disorder and nonclinical population mixed together; Y, reported; RMET, Reading the Mind in the Eye Test; HT, Hinting Task; TASIT-III, The Awareness of Social Inference Test - Part three; FPT, Faux Pas Test; SAT-MC, Social Attribution Task-multiple choice; TSST, The Short Story Task; MASC, Movie for the Assessment of Social Cognition; A-ToM, Adult-Theory of Mind; CST, Comic Strip Task; PST, Picture Sequencing Task; SST, Social shape task; SSFT, Strange Stories film task.

## **Supplementary Table 3.** Meta-regression analyses on internal reliability (Cronbach’s alpha).

| Task | Population | Predictor | k | F | b | SE | p value |
| --- | --- | --- | --- | --- | --- | --- | --- |
| RMET | NC | Age | 29 | 1.302 | 0.006 | 0.005 | 0.264 |
|  |  | gender | 31 | 0.446 | -0.135 | 0.202 | 0.510 |
|  |  | Years of Education | 8 | 0.355 | 0.022 | 0.038 | 0.573 |
|  |  | Sample size | 31 | 0.283 | 0.001 | 0.001 | 0.599 |
|  |  | Mean of task scores | 24 | 0.024 | 0.003 | 0.018 | 0.878 |
|  |  | SD of task scores | 23 | 14.914 | 0.180 | 0.047 | **<0.001** |
|  |  | Continent | 31 | 0.391 | / | / | 0.813 |
|  |  | Quality category | 31 | 1.061 | / | / | 0.360 |
|  |  | Publication year | 31 | 0.047 | -0.002 | 0.009 | 0.830 |
| HT | NC | Age | 8 | 11.004 | 0.015 | 0.005 | **0.016** |
|  |  | gender | 8 | 0.059 | -0.153 | 0.628 | 0.816 |
|  |  | Years of Education | 7 | 0.41 | 0.052 | 0.060 | 0.429 |
|  |  | Sample size | 8 | 2.155 | -0.001 | 0.001 | 0.192 |
|  |  | Mean of task scores | 8 | 2.182 | -0.042 | 0.028 | 0.190 |
|  |  | SD of task scores | 8 | 2.509 | 0.106 | 0.067 | 0.164 |
|  |  | Continent | 8 | 0.878 | / | / | 0.471 |
|  |  | Quality category | 8 | 1.303 | / | / | 0.350 |
|  |  | Publication year | 8 | 0.038 | -0.002 | 0.027 | 0.851 |
|  | SZ | Age | 11 | 1.065 | -0.006 | 0.006 | 0.329 |
|  |  | gender | 13 | 1.116 | 0.568 | 0.538 | 0.313 |
|  |  | Years of Education | 9 | 14.740 | -0.153 | 0.040 | **0.006** |
|  |  | Sample size | 13 | 0.140 | -0.001 | 0.001 | 0.715 |
|  |  | Mean of task scores | 10 | 1.254 | -0.051 | 0.045 | 0.295 |
|  |  | SD of task scores | 10 | 3.931 | 0.131 | 0.066 | 0.083 |
|  |  | Continent | 13 | 0.932 | / | / | 0.464 |
|  |  | Quality category | 13 | 1.203 | / | / | 0.340 |
|  |  | Publication year | 13 | 0.001 | 0.001 | 0.018 | 0.974 |
| TASIT-III | NC | Age | 5 | 46.960 | 0.022 | 0.003 | **0.006** |
|  |  | gender | 5 | 0.001 | -0.006 | 0.814 | 0.995 |
|  |  | Years of Education | 5 | 0.007 | 0.007 | 0.086 | 0.939 |
|  |  | Sample size | 5 | 0.9331 | -0.001 | 0.001 | 0.406 |
|  |  | Mean of task scores | 5 | 9.161 | -0.058 | 0.019 | 0.056 |
|  |  | SD of task scores | 5 | 33.659 | 0.130 | 0.022 | **0.010** |
|  |  | Continent | 5 | 0.060 | / | / | 0.823 |
|  |  | Quality category | 5 | 9.693 | / | / | 0.053 |
|  |  | Publication year | 5 | 1.983 | -0.051 | 0.036 | 0.254 |
|  | SZ | Age | 4 | 0.001 | -0.001 | 0.004 | 0.999 |
|  |  | gender | 5 | 0.188 | -0.093 | 0.214 | 0.694 |
|  |  | Years of Education | 5 | 0.054 | 0.004 | 0.018 | 0.832 |
|  |  | Sample size | 5 | 1.141 | -0.001 | 0.001 | 0.364 |
|  |  | Mean of task scores | 4 | 0.091 | -0.002 | 0.007 | 0.791 |
|  |  | SD of task scores | 4 | 0.090 | 0.019 | 0.063 | 0.792 |
|  |  | Continent | 5 | 0.171 | / | / | 0.707 |
|  |  | Quality category | 5 | 0.310 | / | / | 0.617 |
|  |  | Publication year | 5 | 0.691 | -0.005 | 0.006 | 0.467 |
| FPT | NC | Age | 5 | 2.442 | -0.105 | 0.067 | 0.216 |
|  |  | gender | 5 | 0.002 | -0.158 | 3.286 | 0.965 |
|  |  | Years of Education | 3 | / | / | / | / |
|  |  | Sample size | 5 | 0.129 | 0.001 | 0.002 | 0.743 |
|  |  | Mean of task scores | 3 | / | / | / | / |
|  |  | SD of task scores | 3 | / | / | / | / |
|  |  | Continent | 5 | 3.362 | / | / | 0.377 |
|  |  | Quality category | 5 | 1.469 | / | / | 0.312 |
|  |  | Publication year | 5 | 0.413 | -0.024 | 0.038 | 0.566 |
| TSST | NC | Age | 4 | 0.972 | 0.010 | 0.010 | 0.428 |
|  |  | gender | 4 | 0.010 | -0.090 | 0.922 | 0.931 |
|  |  | Years of Education | 1 | / | / | / | / |
|  |  | Sample size | 4 | 2.419 | 0.002 | 0.001 | 0.260 |
|  |  | Mean of task scores | 3 | / | / | / | / |
|  |  | SD of task scores | 3 | / | / | / | / |
|  |  | Continent | 4 | 6.367 | / | / | 0.095 |
|  |  | Quality category | 4 | 0.320 | / | / | 0.629 |
|  |  | Publication year | 4 | 15.734 | 0.036 | 0.009 | 0.058 |

Note. ASD indicates autism-spectrum disorder; SZ, schizophrenia; NC, nonclinical population; ASD-NC, autism-spectrum disorder and nonclinical population mixed together; SZ-NC, schizophrenia and nonclinical population mixed together; RMET, Reading the Mind in the Eye Test; HT, Hinting Task; TASIT-III, The Awareness of Social Inference Test - Part three; FPT, Faux Pas Test; TSST, The Short Story Task; SD, standard deviation.

## **Supplementary** **Table 4.** Meta-regression analyses on test-retest reliability (Pearson’s r, if not specified).

| Task | Population | Predictor | k | F | b | SE | p value |
| --- | --- | --- | --- | --- | --- | --- | --- |
| RMET | NC | Age | 5 | 0.422 | 0.006 | 0.009 | 0.562 |
|  |  | gender | 5 | 2.334 | -0.670 | 0.438 | 0.224 |
|  |  | Years of Education | 3 | / | / | / | / |
|  |  | Sample size | 5 | 1.278 | 0.001 | 0.001 | 0.340 |
|  |  | Mean of task scores | 5 | 0.680 | -0.047 | 0.057 | 0.470 |
|  |  | SD of task scores | 5 | 0.001 | 0.003 | 0.117 | 0.980 |
|  |  | Continental | 5 | 0.116 | / | / | 0.896 |
|  |  | Quality category | 5 | 0.365 | / | / | 0.588 |
|  |  | Publication year | 5 | 0.444 | 0.014 | 0.021 | 0.553 |
|  |  | Interval days | 5 | 0.943 | -0.012 | 0.012 | 0.403 |
|  | NC (ICC) | Age | 7 | 3.604 | -0.035 | 0.018 | 0.116 |
|  |  | gender | 7 | 2.765 | 0.783 | 0.471 | 0.157 |
|  |  | Years of Education | 3 | / | / | / | / |
|  |  | Sample size | 7 | 0.029 | 0.001 | 0.001 | 0.872 |
|  |  | Mean of task scores | 6 | 0.250 | -0.040 | 0.080 | 0.644 |
|  |  | SD of task scores | 6 | 6.383 | 0.411 | 0.163 | 0.065 |
|  |  | Continental | 7 | 0.375 | / | / | 0.709 |
|  |  | Quality category | 7 | 0.801 | / | / | 0.510 |
|  |  | Publication year | 7 | 1.248 | 0.038 | 0.034 | 0.315 |
|  |  | Interval days | 7 | 1.191 | -0.001 | 0.001 | 0.325 |
|  | SZ | Age | 4 | 1.269 | 0.013 | 0.012 | 0.377 |
|  |  | gender | 4 | 2.041 | -1.416 | 0.991 | 0.289 |
|  |  | Years of Education | 4 | 0.871 | -0.175 | 0.188 | 0.449 |
|  |  | Sample size | 4 | 10.177 | 0.002 | 0.001 | 0.086 |
|  |  | Mean of task scores | 3 | / | / | / | / |
|  |  | SD of task scores | 3 | / |  |  |  |
|  |  | Continental | 4 | / | / | / | / |
|  |  | Quality category | 4 | 0.044 | / | / | 0.822 |
|  |  | Publication year | 4 | 0.068 | 0.039 | 0.149 | 0.819 |
|  |  | Interval days | 4 | 4.715 | -0.016 | 0.009 | 0.162 |
| HT | NC | Age | 4 | 1.462 | -0.013 | 0.011 | 0.350 |
|  |  | gender | 4 | 4.133 | 0.898 | 0.442 | 0.179 |
|  |  | Years of Education | 4 | 5.803 | 0.171 | 0.071 | 0.138 |
|  |  | Sample size | 4 | 0.009 | -0.001 | 0.001 | 0.934 |
|  |  | Mean of task scores | 4 | 0.015 | 0.006 | 0.052 | 0.913 |
|  |  | SD of task scores | 4 | 0.014 | -0.014 | 0.117 | 0.915 |
|  |  | Continental | 4 | 4.404 | / | / | 0.221 |
|  |  | Quality category | 4 | 0.075 | / | / | 0.810 |
|  |  | Publication year | 4 | 0.528 | 0.026 | 0.035 | 0.543 |
|  |  | Interval days | 4 | 7.627 | 0.022 | 0.008 | 0.110 |
|  | SZ | Age | 5 | 0.523 | -0.006 | 0.008 | 0.522 |
|  |  | gender | 5 | 0.572 | 0.535 | 0.707 | 0.504 |
|  |  | Years of Education | 5 | 0.801 | 0.101 | 0.113 | 0.437 |
|  |  | Sample size | 5 | 5.624 | -0.001 | 0.001 | 0.098 |
|  |  | Mean of task scores | 4 | 1.566 | -0.040 | 0.032 | 0.337 |
|  |  | SD of task scores | 4 | 1.108 | 0.076 | 0.073 | 0.403 |
|  |  | Continental | 5 | 4.797 | / | / | 0.309 |
|  |  | Quality category | 5 | 3.572 | / | / | 0.155 |
|  |  | Publication year | 5 | 0.906 | -0.024 | 0.025 | 0.411 |
|  |  | Interval days | 5 | 0.266 | 0.004 | 0.008 | 0.642 |
| TASIT-III | SZ | Age | 5 | 0.581 | 0.007 | 0.009 | 0.501 |
|  |  | gender | 5 | 2.057 | -1.265 | 0.882 | 0.247 |
|  |  | Years of Education | 5 | 7.585 | -0.296 | 0.107 | 0.070 |
|  |  | Sample size | 5 | 0.017 | 0.001 | 0.001 | 0.903 |
|  |  | Mean of task scores | 4 | 2.105 | -0.044 | 0.031 | 0.284 |
|  |  | SD of task scores | 4 | 1.809 | 0.287 | 0.213 | 0.311 |
|  |  | Continental | 5 | 8.784 | / | / | 0.067 |
|  |  | Quality category | 5 | 2.100 | / | / | 0.243 |
|  |  | Publication year | 5 | 0.201 | -0.022 | 0.048 | 0.684 |
|  |  | Interval days | 5 | 0.559 | 0.001 | 0.001 | 0.509 |

Note. SZ, schizophrenia; NC, nonclinical population; RMET, Reading the Mind in the Eye Test; HT, Hinting Task; TASIT-III, The Awareness of Social Inference Test - Part three; ICC, intraclass correlation; SD, standard deviation.

## **Supplementary Table 5.** Quality assessment of the included studies**.**

| Study | Item 1 | Item 2 | Item 3 | Item 4 | Item 5 | Item 6 | Overall |
| --- | --- | --- | --- | --- | --- | --- | --- |
| Achim et al., 2012 | V | A | V | A | N/A | N/A | A |
| Adler et al., 2010 | A | A | V | A | N/A | N/A | A |
| Ali & Chamorro-Premuzic, 2010 | A | V | V | A | N/A | N/A | A |
| Andersen et al., 2022 | V | V | V | V | N/A | N/A | V |
| Aykan & Nalçacı, 2018 | V | V | V | V | N/A | N/A | V |
| Bechi et al., 2012 | V | V | V | A | N/A | N/A | A |
| Bedwell et al., 2014 | A | A | V | A | N/A | N/A | A |
| Bell et al., 2010 | V | V | V | V | N/A | N/A | V |
| Bozikas et al., 2011 | V | V | V | A | N/A | N/A | A |
| Brewer et al., 2017 | V | V | V | V | V | V | V |
| Brown et al., 2019 | V | V | V | V | N/A | N/A | V |
| Brown et al., 2023 | V | V | V | V | N/A | N/A | V |
| Brunet-Gouet et al., 2021 | V | V | V | A | N/A | N/A | A |
| Canty et al., 2015 | V | V | V | V | D | V | D |
| Charernboon et al., 2017 | V | V | V | V | D | V | D |
| Chen et al., 2017 | V | V | V | V | A | V | A |
| Cruz et al., 2022 | V | V | V | V | N/A | N/A | V |
| Davidson et al., 2018 | V | A | V | V | D | V | D |
| Dehning et al., 2012 | V | A | V | A | N/A | N/A | A |
| Dodell-Feder et al., 2013 | V | V | V | V | N/A | N/A | V |
| Dziobek et al., 2006 | V | V | V | V | A | V | A |
| Faísca et al., 2016 | V | V | V | V | N/A | N/A | V |
| Fekete et al., 2022 | V | V | V | V | A | V | A |
| Ferguson et al., 2010 | V | V | V | A | N/A | N/A | A |
| Fernández-Abascal et al., 2013 | V | V | V | V | A | V | A |
| Fernández-Modamio et al., 2018 | V | V | V | V | A | V | A |
| Fossati et al., 2018 | V | V | V | V | N/A | N/A | V |
| Frøyhaug et al., 2019 | V | V | V | V | N/A | N/A | V |
| Gil et al., 2012 | V | V | V | V | D | V | D |
| Giordano et al., 2019 | V | V | V | V | N/A | N/A | V |
| Girli et al., 2014 | V | V | V | V | N/A | N/A | V |
| Gong et al., 2014 | V | V | V | A | N/A | N/A | A |
| Gourlay et al., 2020 | V | V | V | V | N/A | N/A | V |
| Han et al., 2020 | V | V | V | A | N/A | N/A | A |
| Harkness et al., 2010 | V | V | V | A | N/A | N/A | A |
| Higgins et al., 2023 | V | V | V | V | N/A | N/A | V |
| Horan et al., 2012 | V | V | V | A | D | V | D |
| Huang et al., 2023 | V | V | V | V | A | V | A |
| Isernia et al., 2023 | V | V | V | V | N/A | N/A | V |
| Jankowiak-Suida et al., 2016 | V | V | V | V | D | V | D |
| Jarvers et al., 2022 | V | V | V | V | N/A | N/A | V |
| Johannesen et al., 2013 | V | V | V | V | N/A | N/A | V |
| Johannesen et al., 2018 | V | A | V | V | D | V | D |
| Khorashad et al., 2015 | V | V | V | V | D | V | D |
| Klein et al., 2020 | V | V | V | V | D | V | D |
| Klein et al., 2022 | V | V | V | V | N/A | N/A | V |
| Koo et al., 2020 | V | V | V | V | V | V | V |
| Krawczyk et al., 2020 | V | V | V | V | N/A | N/A | V |
| Lahera et al., 2016 | V | A | V | V | A | V | A |
| Le Donne et al., 2023 | V | V | V | V | N/A | N/A | V |
| Lee et al., 2018 | V | V | V | V | N/A | N/A | V |
| Lee et al., 2020 | V | A | V | V | D | V | D |
| Lim et al., 2020 | V | A | V | V | V | V | A |
| Lima-Sánchez et al., 2019 | V | A | V | V | D | V | D |
| Livingston et al., 2021 | V | V | V | V | N/A | N/A | V |
| Ludwig et al., 2017 | V | A | V | V | V | V | A |
| Mallawaarachchi et al., 2019 | V | V | V | V | N/A | N/A | V |
| Mar et al., 2006 | V | A | V | A | N/A | N/A | A |
| Megías-Robles et al., 2020 | V | V | V | A | N/A | N/A | A |
| Melchers et al., 2015 | A | A | V | A | N/A | N/A | A |
| Morrison et al., 2019 | V | V | V | V | N/A | N/A | V |
| Murray et al., 2017 | V | V | V | V | N/A | N/A | V |
| Navarro et al., 2022 | V | V | V | V | N/A | N/A | V |
| Negrao et al., 2016 | V | V | V | V | N/A | N/A | V |
| Ng et al., 2015 | V | V | V | A | N/A | N/A | A |
| Pallathra et al., 2018 | V | V | V | A | N/A | N/A | A |
| Park et al., 2018 | V | V | V | A | N/A | N/A | A |
| Pfaltz et al., 2013 | V | V | V | V | A | V | A |
| Pinkham et al., 2016 | V | V | V | V | A | V | A |
| Pinkham et al., 2018 | V | V | V | V | A | V | A |
| Preller et al., 2015 | V | A | V | A | N/A | N/A | A |
| Prevost et al., 2014 | V | V | V | V | N/A | N/A | V |
| Roberts et al., 2009 | V | V | V | V | N/A | N/A | V |
| Sandor et al., 2023 | V | V | V | V | N/A | N/A | V |
| Sedgwick et al., 2021 | V | V | V | A | N/A | N/A | A |
| Serra-Mayoral et al., 2021 | V | V | V | V | N/A | N/A | V |
| Smeets et al., 2009 | A | A | V | A | N/A | N/A | A |
| Söderstrand et al., 2012 | V | V | V | V | N/A | N/A | V |
| Thibaudeau et al., 2018 | V | A | V | V | V | V | A |
| Thoma et al., 2014 | A | A | V | A | N/A | N/A | A |
| Turner et al., 2020 | V | V | V | A | N/A | N/A | A |
| Van Doesum et al., 2013 | V | V | V | A | N/A | N/A | A |
| Vellante et al., 2013 | V | V | V | V | D | V | D |
| Vidarsdottir et al., 2019 | V | V | V | A | N/A | N/A | A |
| Voracek et al., 2005 | V | V | V | V | N/A | N/A | V |
| Watanabe et al., 2021 | V | V | V | V | N/A | N/A | V |
| Yeh et al., 2023 | V | V | V | V | D | V | D |
| Yildirim et al., 2011 | V | V | V | V | D | V | D |
| Zhu et al., 2007 | V | A | V | A | D | V | D |
| Zıvralı Yarar et al., 2021 | V | V | V | A | N/A | N/A | A |

Note. V indicates very good; A, adequate; D, doubtful Item 1: “Was a Cronbach's alpha, Mcdonal's omega, Kappa, intraclass correlation coefficient (ICC), or Pearson’s r correlation calculated and reported?”; Item 2: “Was the internal consistency or test-reliability statistic calculated for each unidimensional scale or subscale separately?”; Item 3: “Were the test conditions similar for the measurements? e.g. type of administration, environment, instructions”; Item 4: “Were there any other important flaws in the design or statistical methods of the study?”; Item 5 (For studies reported test-retest reliability only): “Was the time interval appropriate and reported?”; Item 6 (For studies reported test-retest reliability only): “Were participants stable in the interim period on the construct to be measured?”

## **Supplementary Figure 1.** A summary of the risk-of-bias evaluation.

**
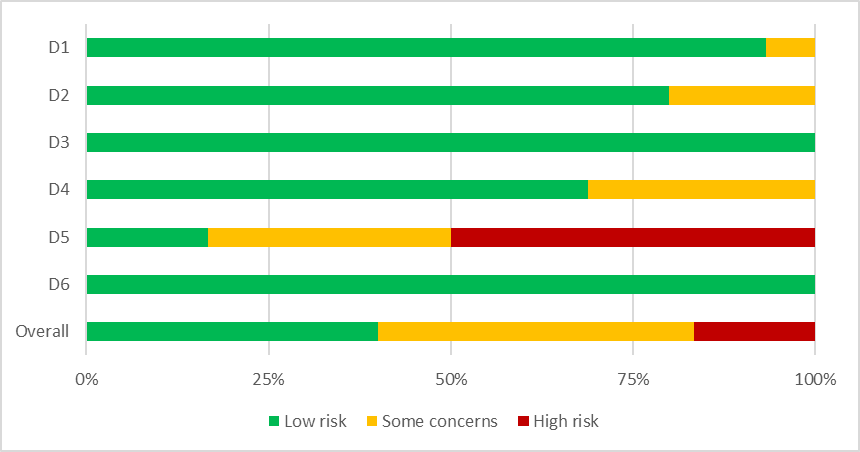
**

Note. Item 1: “Was a Cronbach's alpha, Mcdonal's omega, Kappa, intraclass correlation coefficient (ICC), or Pearson’s r correlation calculated and reported?”; Item 2: “Was the internal consistency or test-reliability statistic calculated for each unidimensional scale or subscale separately?”; Item 3: “Were the test conditions similar for the measurements? e.g. type of administration, environment, instructions”; Item 4: “Were there any other important flaws in the design or statistical methods of the study?”; Item 5 (For studies reported test-retest reliability only): “Was the time interval appropriate and reported?”; Item 6 (For studies reported test-retest reliability only): “Were participants stable in the interim period on the construct to be measured?”

## **Supplementary Figure 2.** Funnel plots of the meta-analyses of internal consistency.

a) Internal consistency of RMET in NC b) Internal consistency of RMET in SZ


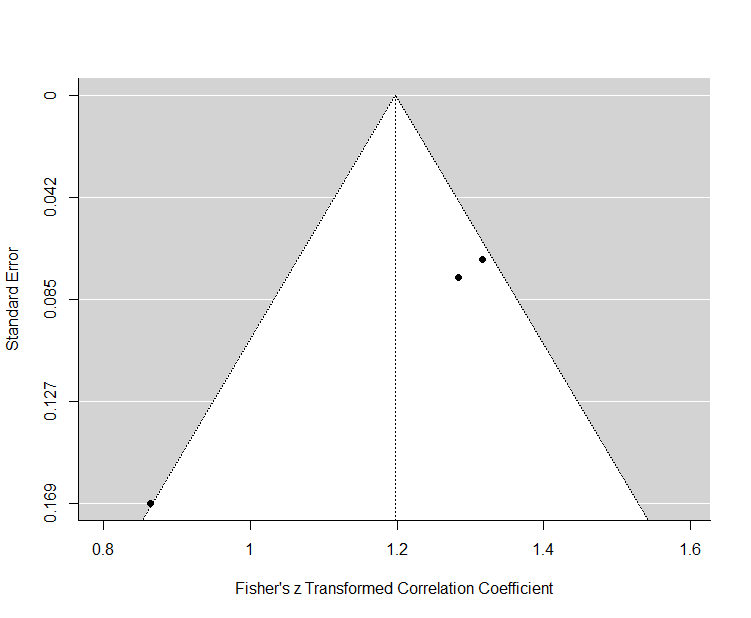

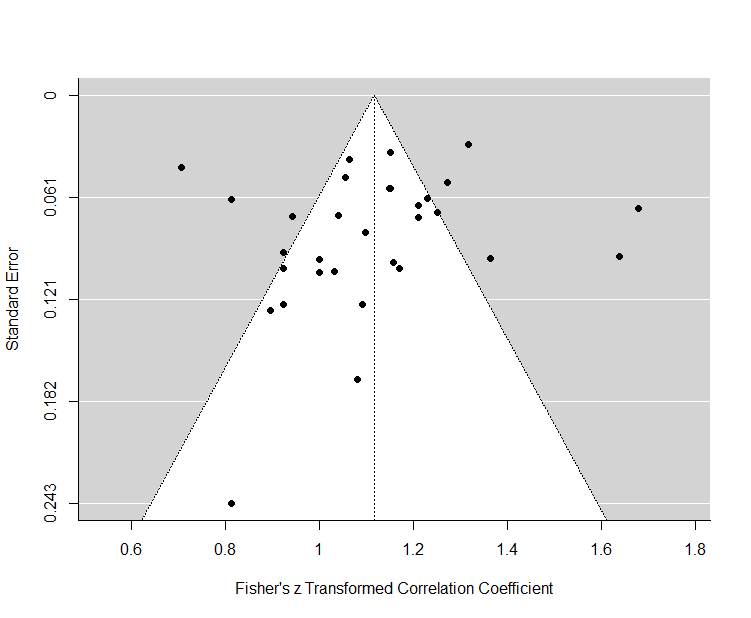


c) Internal consistency of RMET in SZ-NC d) Internal consistency of HT in NC


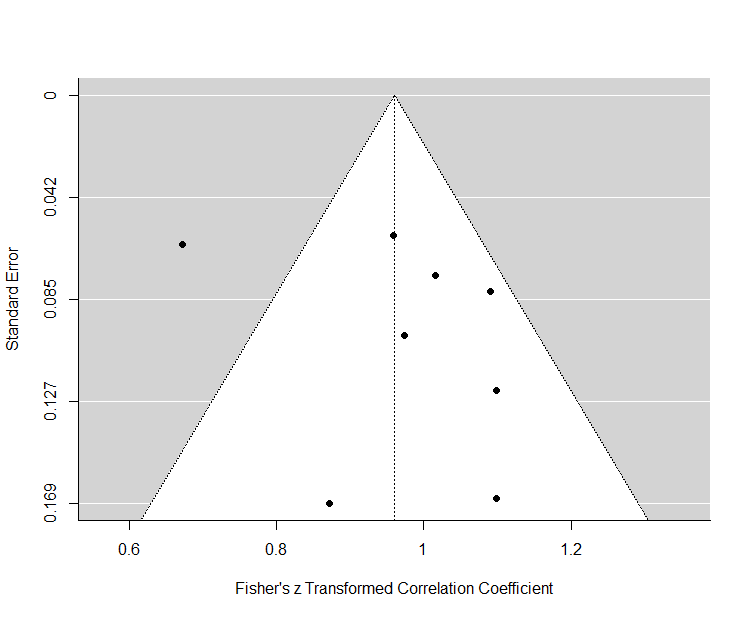

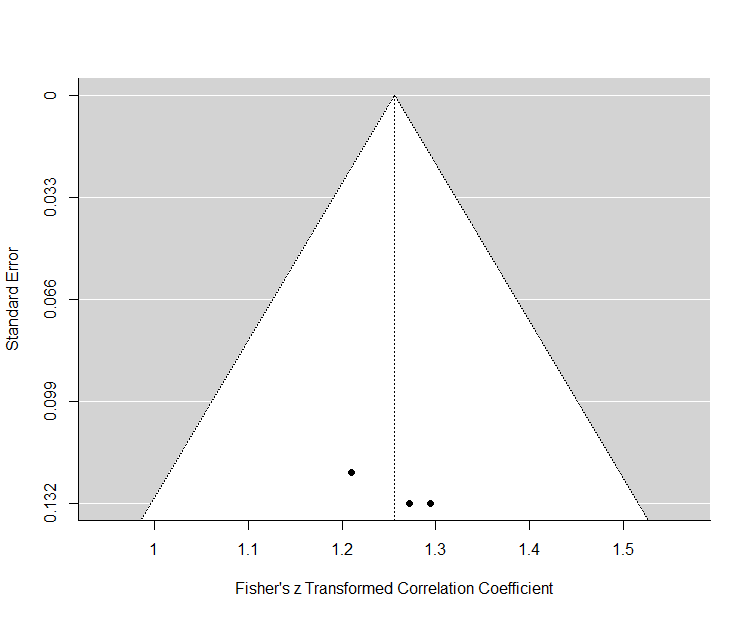


e) Internal consistency of HT in SZ f) Internal consistency of HT in SZ-NC


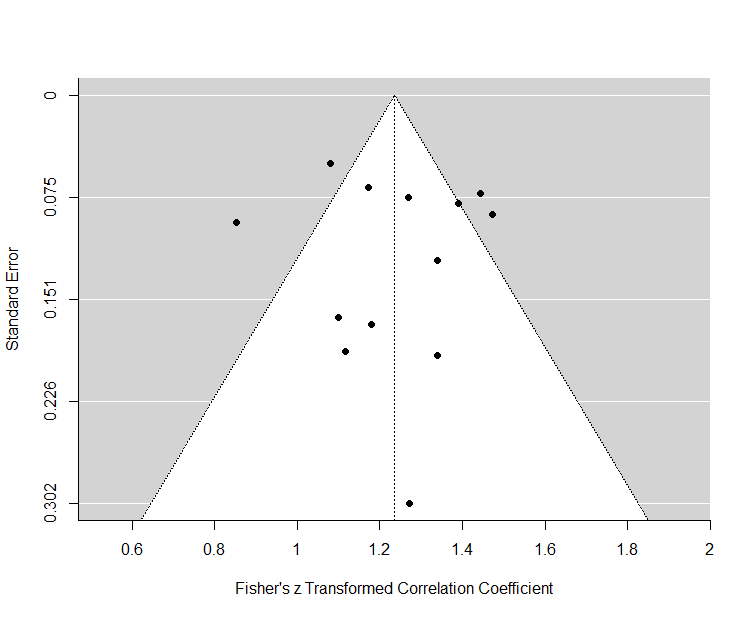

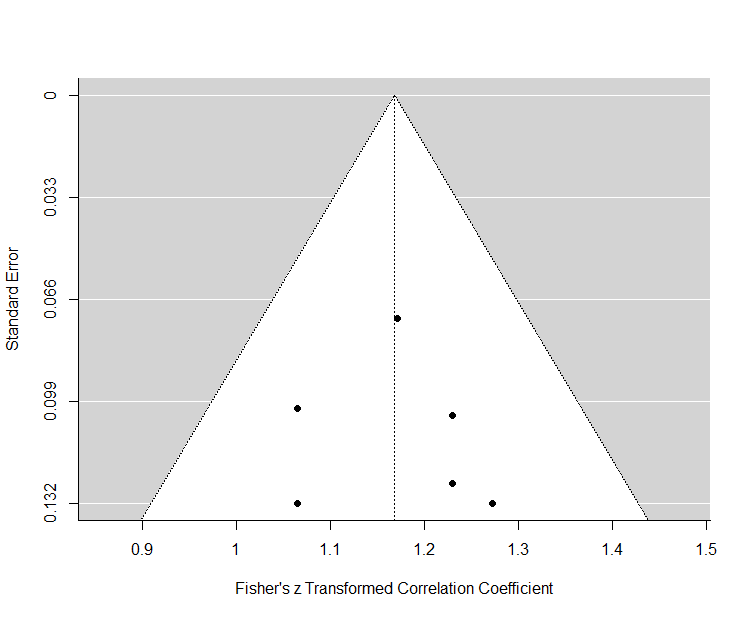


g) Internal consistency of TASIT-III in NC h) Internal consistency of TASIT-III in SZ


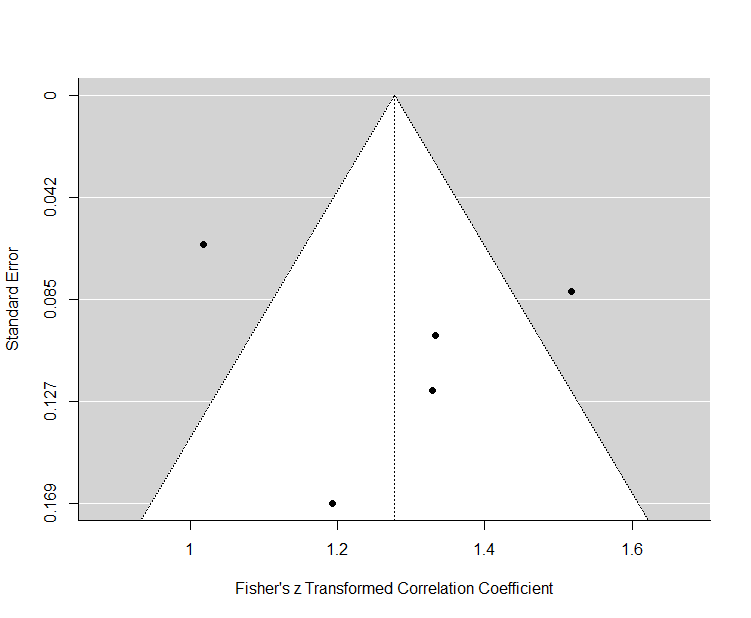

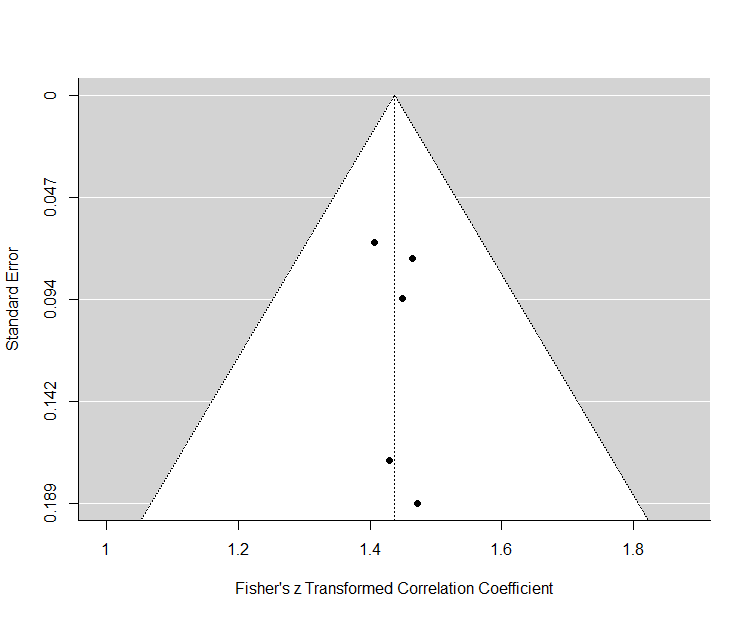


i) Internal consistency of SAT-MC in NC j) Internal consistency of SAT-MC in SZ


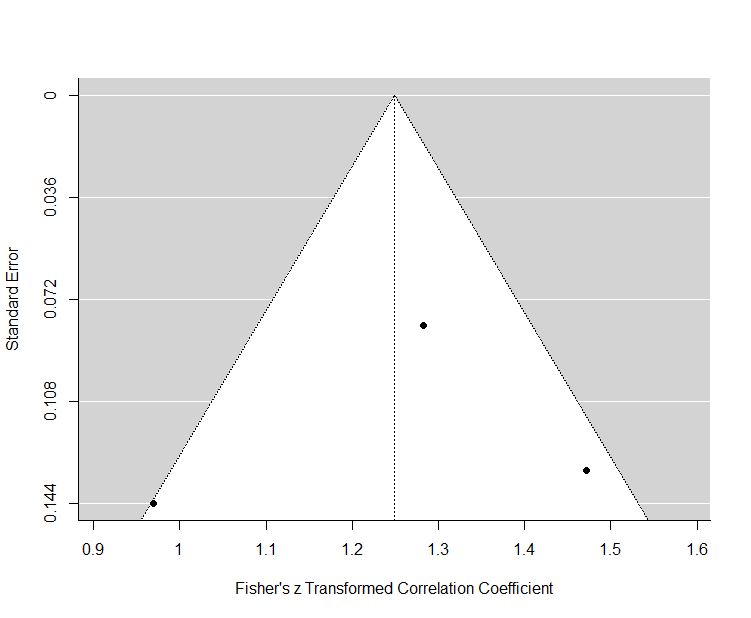

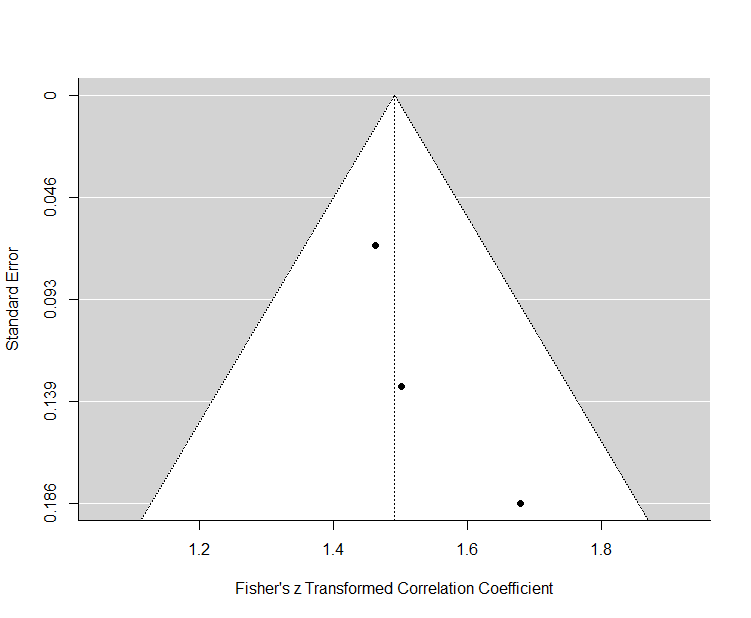


i) Internal consistency of FPT in NC j) Internal consistency of TSST in NC


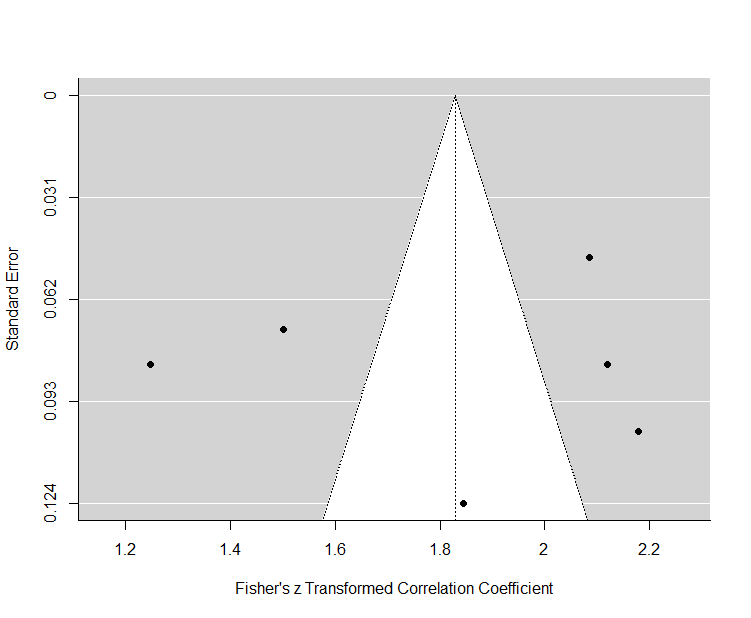

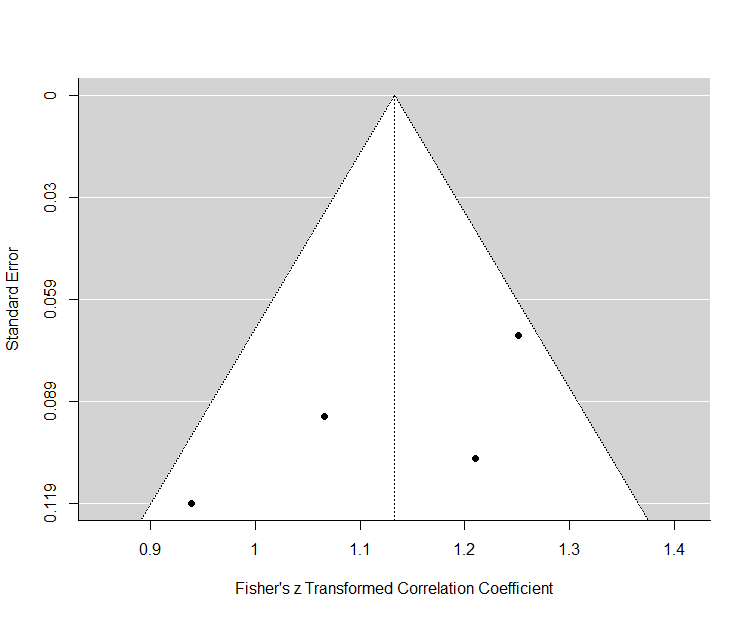


## **Supplementary Figure 3.** Funnel plots of the meta-analyses of test-retest reliability.

a) Test-retest reliability of RMET in NC using Pearson’s r b) Test-retest reliability of RMET in NC using ICC


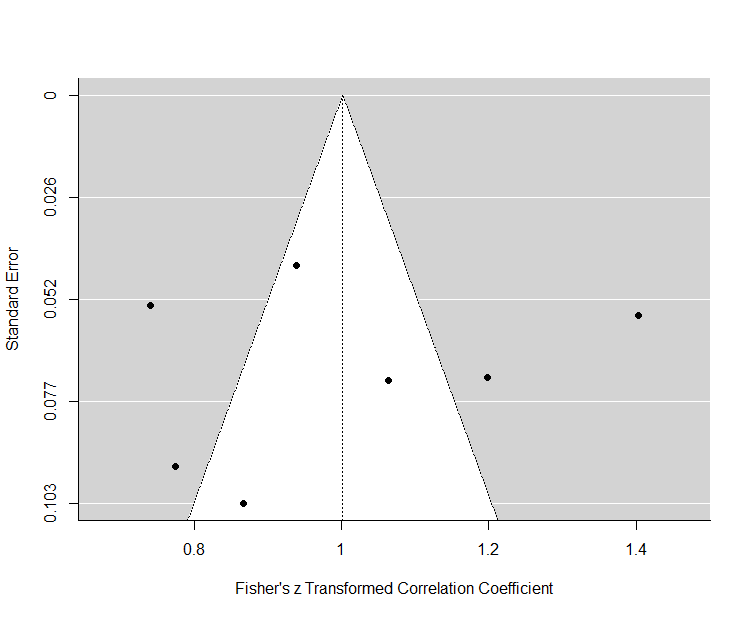

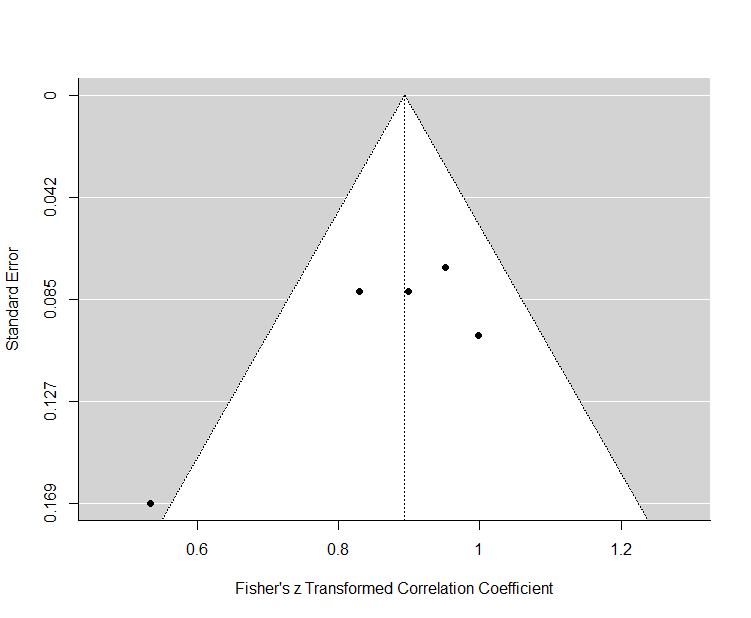


c) Test-retest reliability of RMET in SZ d) Test-retest reliability of HT in NC


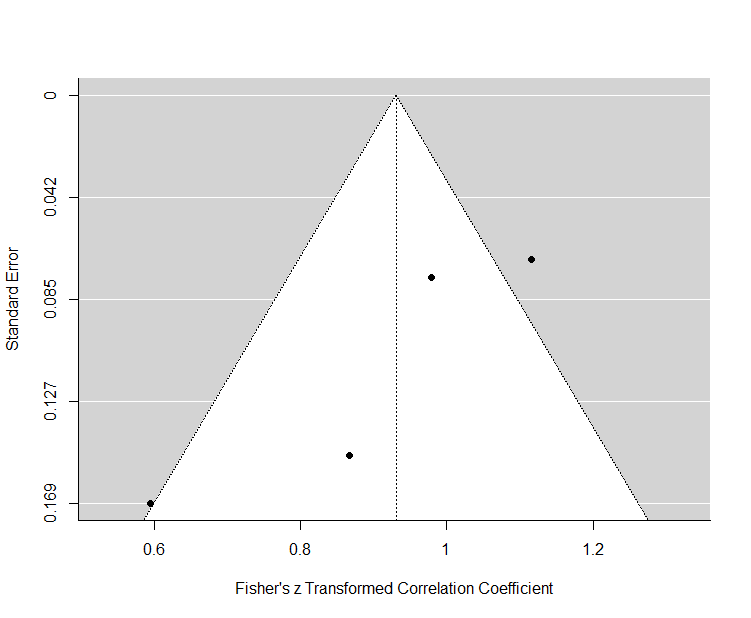

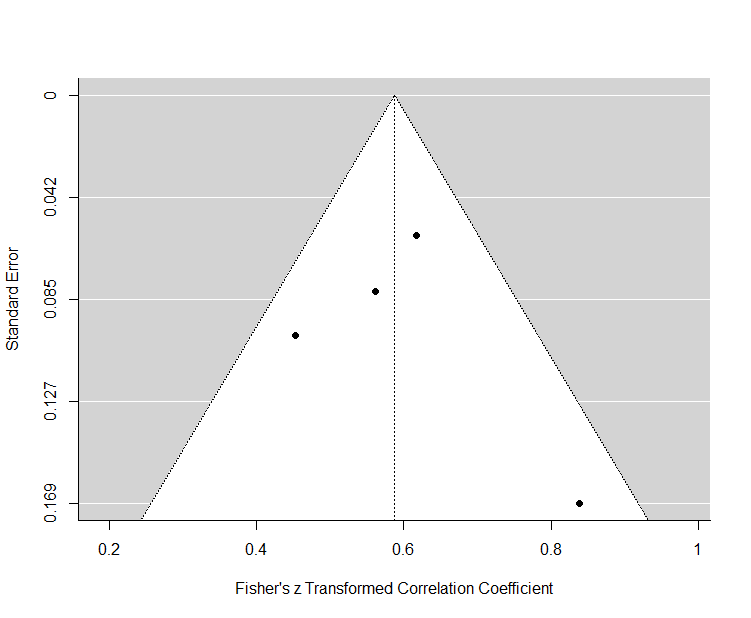


e) Test-retest reliability of HT in SZ f) Test-retest reliability of TASIT-III in NC


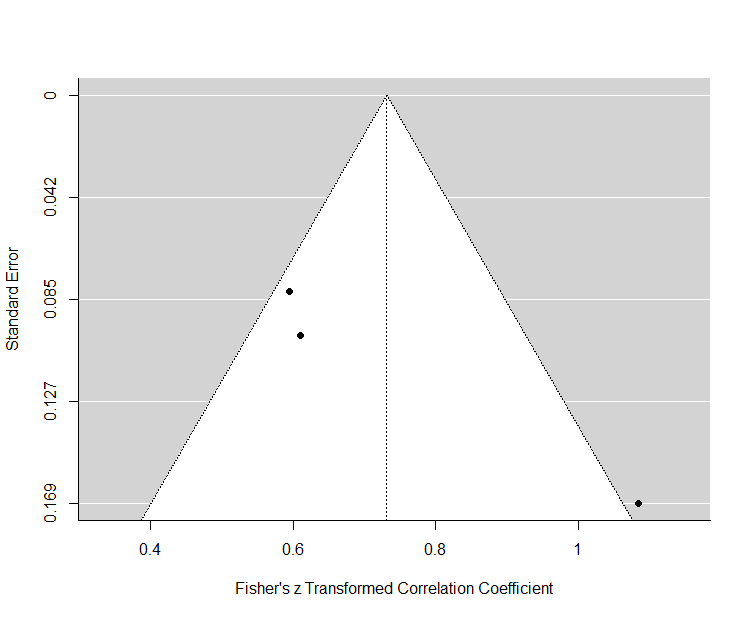

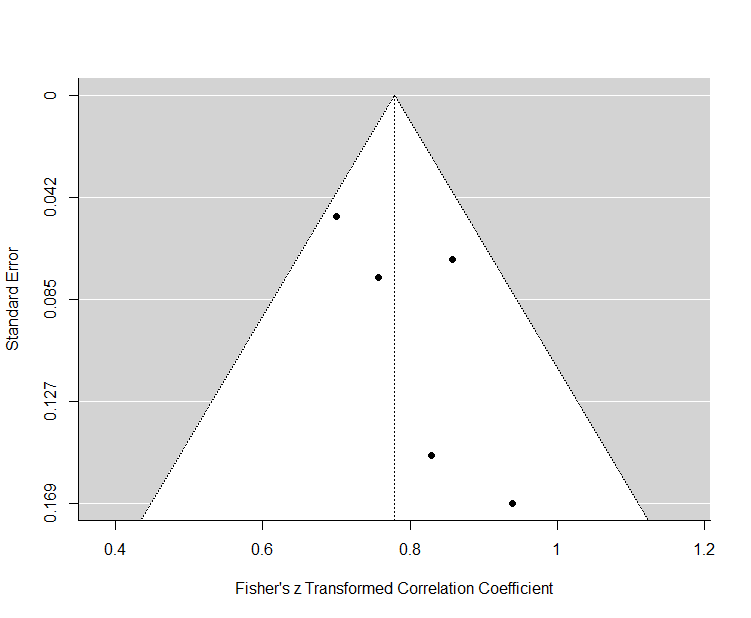


g) Test-retest reliability of TASIT-III in SZ


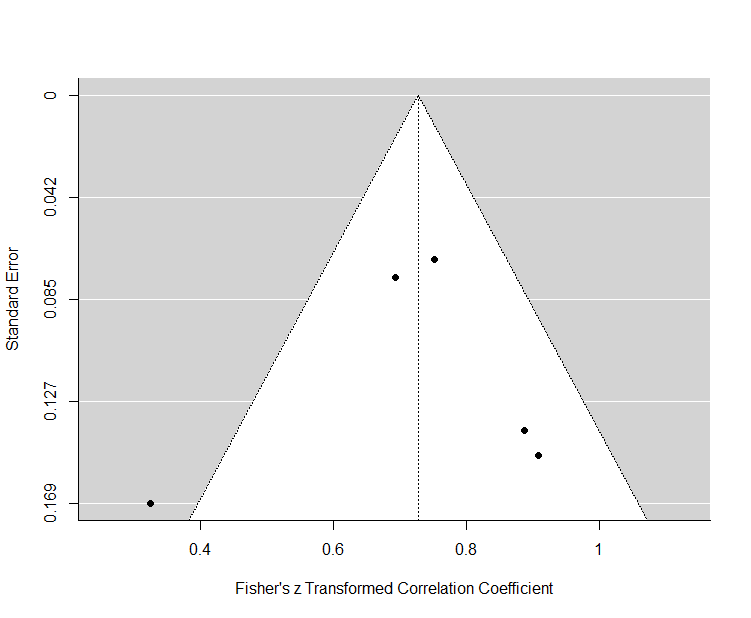


## References of included studies

Achim, A. M., Ouellet, R., Roy, M.-A., & Jackson, P. L. (2012). Mentalizing in first-episode psychosis. *Psychiatry Research*, *196*(2-3), 207–213. https://doi.org/10.1016/j.psychres.2011.10.011

Adler, N., Nadler, B., Eviatar, Z., & Shamay-Tsoory, S. G. (2010). The relationship between theory of mind and autobiographical memory in high-functioning autism and Asperger syndrome. *Psychiatry Research*, *178*(1), 214–216. https://doi.org/10.1016/j.psychres.2009.11.015

Ali, F., & Chamorro-Premuzic, T. (2010). Investigating Theory of Mind deficits in nonclinical psychopathy and Machiavellianism. *Personality and Individual Differences*, *49*(3), 169–174. https://doi.org/10.1016/j.paid.2010.03.027

Andersen, N. K., Rimvall, M. K., Jeppesen, P., Bentz, M., Jepsen, J. R. M., Clemmensen, L., et al. (2022). A psychometric investigation of the multiple-choice version of Animated Triangles Task to measure Theory of Mind in adolescence. *PLOS ONE*, *17*(3), e0264319–e0264319. https://doi.org/10.1371/journal.pone.0264319

Aykan, S., & Nalçacı, E. (2018). Assessing Theory of Mind by Humor: The Humor Comprehension and Appreciation Test (ToM-HCAT). *Frontiers in Psychology*, *9*, 1470. https://doi.org/10.3389/fpsyg.2018.01470

Bechi, M., Riccaboni, R., Ali, S., Fresi, F., Buonocore, M., Bosia, M., et al. (2012). Theory of mind and emotion processing training for patients with schizophrenia: Preliminary findings. *Psychiatry Research*, *198*(3), 371–377. https://doi.org/10.1016/j.psychres.2012.02.004

Bedwell, J. S., Compton, M. T., Jentsch, F. G., Deptula, A. E., Goulding, S. M., & Tone, E. B. (2014). Latent Factor Modeling of Four Schizotypy Dimensions with Theory of Mind and Empathy. *PLoS ONE*, *9*(11), e113853. https://doi.org/10.1371/journal.pone.0113853

Bell, M. D., Fiszdon, J. M., Greig, T. C., & Wexler, B. E. (2010). Social attribution test — multiple choice (SAT-MC) in schizophrenia: Comparison with community sample and relationship to neurocognitive, social cognitive and symptom measures. *Schizophrenia Research*, *122*(1-3), 164–171. https://doi.org/10.1016/j.schres.2010.03.024

Bozikas, V. P., Giannakou, M., Kosmidis, M. H., Kargopoulos, P., Kioseoglou, G., Liolios, D., & Garyfallos, G. (2011). Insights into theory of mind in schizophrenia: The impact of cognitive impairment. *Schizophrenia Research*, *130*(1-3), 130–136. https://doi.org/10.1016/j.schres.2011.04.025

Brewer, N., Young, R. L., & Barnett, E. (2017). Erratum to: Measuring Theory of Mind in Adults with Autism Spectrum Disorder. *Journal of Autism and Developmental Disorders*, *47*(7), 1942–1943. https://doi.org/10.1007/s10803-017-3150-0

Brown, M. I., Heck, P. R., & Chabris, C. F. (2023). The Social Shapes Test as a Self-Administered, Online Measure of Social Intelligence: Two Studies with Typically Developing Adults and Adults with Autism Spectrum Disorder. *Journal of Autism and Developmental Disorders*, 1–16. https://doi.org/10.1007/s10803-023-05901-2

Brown, M. I., Ratajska, A., Hughes, S. L., Fishman, J. B., Huerta, E., & Chabris, C. F. (2019). The social shapes test: A new measure of social intelligence, mentalizing, and theory of mind. *Personality and Individual Differences*, *143*, 107–117. https://doi.org/10.1016/j.paid.2019.01.035

Brunet-Gouet, E., Decaix-Tisserand, C., Urbach, M., Bazin, N., Aouizerate, B., Brunel, L., et al. (2021). Outcome prediction with a social cognitive battery: a multicenter longitudinal study. *npj Schizophrenia*, *7*(1). https://doi.org/10.1038/s41537-021-00160-5

Canty, A. L., Neumann, D. L., Fleming, J., & Shum, D. H. K. (2015). Evaluation of a newly developed measure of theory of mind: The virtual assessment of mentalising ability. *Neuropsychological Rehabilitation*, *27*(5), 834–870. https://doi.org/10.1080/09602011.2015.1052820

Charernboon, T., & Lerthattasilp, T. (2017). The Reading the Mind in the Eyes Test: Validity and Reliability of the Thai Version. *Cognitive and Behavioral Neurology*, *30*(3), 98–101. https://doi.org/10.1097/wnn.0000000000000130

Chen, K.-W., Lee, S.-C., Chiang, H.-Y., Syu, Y.-C., Yu, X.-X., & Hsieh, C.-L. (2017). Psychometric properties of three measures assessing advanced theory of mind: Evidence from people with schizophrenia. *Psychiatry Research*, *257*, 490–496. https://doi.org/10.1016/j.psychres.2017.08.026

Cruz, B. F., Oliveira, A. M. de, Del-Ben, C. M., Corcoran, R., & Salgado, J. V. (2022). Validation of the Brazilian version of the Hinting Task and Facial Emotion Recognition Test (FERT-100) in patients with schizophrenia. *Dementia & Neuropsychologia*, *16*(3), 300–308. https://doi.org/10.1590/1980-5764-dn-2021-0108

Davidson, C. A., Lesser, R., Parente, L. T., & Fiszdon, J. M. (2018). Psychometrics of social cognitive measures for psychosis treatment research. *Schizophrenia Research*, *193*, 51–57. https://doi.org/10.1016/j.schres.2017.06.018

Dehning, S., Girma, E., Gasperi, S., Meyer, S., Tesfaye, M., & Siebeck, M. (2012). Comparative cross-sectional study of empathy among first year and final year medical students in Jimma University, Ethiopia: Steady state of the heart and opening of the eyes. *BMC Medical Education*, *12*(1). https://doi.org/10.1186/1472-6920-12-34

Dodell-Feder, D., Lincoln, S. H., Coulson, J. P., & Hooker, C. I. (2013). Using Fiction to Assess Mental State Understanding: A New Task for Assessing Theory of Mind in Adults. *PLoS ONE*, *8*(11), e81279. https://doi.org/10.1371/journal.pone.0081279

Dziobek, I., Fleck, S., Kalbe, E., Rogers, K., Hassenstab, J., Brand, M., et al. (2006). Introducing MASC: A Movie for the Assessment of Social Cognition. *Journal of Autism and Developmental Disorders*, *36*(5), 623–636. https://doi.org/10.1007/s10803-006-0107-0

Faísca, L., Afonseca, S., Brüne, M., Gonçalves, G., Gomes, A., & Martins, A. T. (2016). Portuguese Adaptation of a Faux Pas Test and a Theory of Mind Picture Stories Task. *Psychopathology*, *49*(3), 143–152. https://doi.org/10.1159/000444689

Fekete, Z., Vass, E., Balajth, R., Tana, Ü., Nagy, A. C., Domján, N., et al. (2022). Regrouping scalets: Psychometric properties of the theory of mind picture stories task in a schizophrenic sample. *Neuropsychological Rehabilitation*, *32*(9), 2227–2247. https://doi.org/10.1080/09602011.2021.1930559

Ferguson, F. J., & Austin, E. J. (2010). Associations of trait and ability emotional intelligence with performance on Theory of Mind tasks in an adult sample. *Personality and Individual Differences*, *49*(5), 414–418. https://doi.org/10.1016/j.paid.2010.04.009

Fernández-Abascal, E. G., Cabello, R., Fernández-Berrocal, P., & Baron-Cohen, S. (2013). Test-retest reliability of the “Reading the Mind in the Eyes” test: a one-year follow-up study. *Molecular Autism*, *4*(1). https://doi.org/10.1186/2040-2392-4-33

Fernández-Modamio, M., Arrieta-Rodríguez, M., Bengochea-Seco, R., Santacoloma-Cabero, I., Gómez de Tojeiro-Roce, J., García-Polavieja, B., et al. (2018). Faux-Pas Test: A Proposal of a Standardized Short Version. *Clinical Schizophrenia & Related Psychoses*, 10.3371/CSRP.FEAR.061518. https://doi.org/10.3371/csrp.fear.061518

Fossati, A., Borroni, S., Dziobek, I., Fonagy, P., & Somma, A. (2018). Thinking about assessment: Further evidence of the validity of the Movie for the Assessment of Social Cognition as a measure of mentalistic abilities. *Psychoanalytic Psychology*, *35*(1), 127–141. https://doi.org/10.1037/pap0000130

Frøyhaug, M., Andersson, S., Andreassen, O. A., Ueland, T., & Vaskinn, A. (2019). Theory of mind in schizophrenia and bipolar disorder: psychometric properties of the Norwegian version of the Hinting Task. *Cognitive Neuropsychiatry*, *24*(6), 454–469. https://doi.org/10.1080/13546805.2019.1674645

Gil, D., Fernández-Modamio, M., Bengochea, R., & Arrieta, M. (2012). Adaptation of the Hinting Task theory of the mind test to Spanish. *Revista de Psiquiatría y Salud Mental (English Edition)*, *5*(2), 79–88. https://doi.org/10.1016/j.rpsmen.2011.11.002

Giordano, M., Licea-Haquet, G., Navarrete, E., Valles-Capetillo, E., Lizcano-Cortés, F., Carrillo-Peña, A., & Zamora-Ursulo, A. (2019). Comparison between the Short Story Task and the Reading the Mind in the Eyes Test for evaluating Theory of Mind: A replication report. *Cogent Psychology*, *6*(1). https://doi.org/10.1080/23311908.2019.1634326

Girli, A. (2014). Psychometric Properties of the Turkish Child and Adult Form of “Reading the Mind in the Eyes Test.” *Psychology*, *05*(11), 1321–1337. https://doi.org/10.4236/psych.2014.511143

Gong, P., Liu, J., Li, S., & Zhou, X. (2013). Dopamine beta-hydroxylase gene modulates individuals’ empathic ability. *Social Cognitive and Affective Neuroscience*, *9*(9), 1341–1345. https://doi.org/10.1093/scan/nst122

Gourlay, C., Collin, P., Caron, P.-O., D’Auteuil, C., & Scherzer, P. B. (2020). Psychometric assessment of social cognitive tasks. *Applied Neuropsychology: Adult*, *29*(4), 731–749. https://doi.org/10.1080/23279095.2020.1807348

Han, M., & Jun, S. S. (2020). Effects of Psychotic Symptoms and Social Cognition on Job Retention in Patients with Schizophrenia in Korea. *International Journal of Environmental Research and Public Health*, *17*(8), 2628. https://doi.org/10.3390/ijerph17082628

Harkness, K. L., Jacobson, J. A., Duong, D., & Sabbagh, M. A. (2010). Mental state decoding in past major depression: Effect of sad versus happy mood induction. *Cognition & Emotion*, *24*(3), 497–513. https://doi.org/10.1080/02699930902750249

Higgins, W. C., Ross, R. M., Langdon, R., & Polito, V. (2022). The “Reading the Mind in the Eyes” Test Shows Poor Psychometric Properties in a Large, Demographically Representative U.S. Sample. *Assessment*, *30*(6), 1777–1789. https://doi.org/10.1177/10731911221124342

Horan, W. P., Green, M. F., DeGroot, M., Fiske, A., Hellemann, G., Kee, K., et al. (2012). Social Cognition in Schizophrenia, Part 2: 12-Month Stability and Prediction of Functional Outcome in First-Episode Patients. *Schizophrenia Bulletin*, *38*(4), 865–872. https://doi.org/10.1093/schbul/sbr001

Huang, Y.-L., Chen, T.-T., & Tseng, H.-H. (2023). Mentalizing in a Movie for the Assessment of Social Cognition (MASC) : The Validation in a Taiwanese Sample. *BMC psychology*, *11*(1), 287. https://doi.org/10.21203/rs.3.rs-2650269/v1

Isernia, S., Rossetto, F., Shamay-Tsoory, S., Marchetti, A., & Baglio, F. (2023). Standardization and normative data of the 48-item Yoni short version for the assessment of theory of mind in typical and atypical conditions. *Frontiers in Aging Neuroscience*, *14*, 1048599. https://doi.org/10.3389/fnagi.2022.1048599

Jankowiak-Siuda, K., Baron-Cohen, S., Białaszek, W., Dopierała, A., Kozłowska, A., & Rymarczyk, K. (2016). PSYCHOMETRIC EVALUATION OF THE “READING THE MIND IN THE EYES” TEST WITH SAMPLES OF DIFFERENT AGES FROM A POLISH POPULATION. *Studia Psychologica*, *58*(1), 18–31. https://doi.org/10.21909/sp.2016.01.704

Jarvers, I., Döhnel, K., Blaas, L., Ullmann, M., Langguth, B., Rupprecht, R., & Sommer, M. (2023). “Why do they do it?”: The short‐story task for measuring fiction‐based mentalizing in autistic and non‐autistic individuals. *Autism Research*, *16*(3), 558–568. https://doi.org/10.1002/aur.2871

Johannesen, J. K., Fiszdon, J. M., Weinstein, A., Ciosek, D., & Bell, M. D. (2018). The Social Attribution Task - Multiple Choice (SAT-MC): Psychometric comparison with social cognitive measures for schizophrenia research. *Psychiatry Research*, *262*, 154–161. https://doi.org/10.1016/j.psychres.2018.02.011

Johannesen, J. K., Lurie, J. B., Fiszdon, J. M., & Bell, M. D. (2013). The Social Attribution Task-Multiple Choice (SAT-MC): A Psychometric and Equivalence Study of an Alternate Form. *ISRN Psychiatry*, *2013*, 1–9. https://doi.org/10.1155/2013/830825

Khorashad, B. S., Baron-Cohen, S., Roshan, G. M., Kazemian, M., Khazai, L., Aghili, Z., et al. (2015). The “Reading the Mind in the Eyes” Test: Investigation of Psychometric Properties and Test–Retest Reliability of the Persian Version. *Journal of Autism and Developmental Disorders*, *45*(9), 2651–2666. https://doi.org/10.1007/s10803-015-2427-4

Klein, H. S., Springfield, C. R., Bass, E., Ludwig, K., Penn, D. L., Harvey, P. D., & Pinkham, A. E. (2020). Measuring mentalizing: A comparison of scoring methods for the hinting task. *International Journal of Methods in Psychiatric Research*, *29*(2). https://doi.org/10.1002/mpr.1827

Klein, H., Springfield, C. R., & Pinkham, A. E. (2022). Measuring social cognition within the university: The Social Cognition Psychometric Evaluation (SCOPE) battery in an undergraduate sample. *Applied Neuropsychology: Adult*, 1–8. https://doi.org/10.1080/23279095.2022.2082875

Koo, S. J., Kim, Y. J., Han, J. H., Seo, E., Park, H. Y., Bang, M., et al. (2021). “Reading the Mind in the Eyes Test”: Translated and Korean Versions. *Psychiatry Investigation*, *18*(4), 295–303. https://doi.org/10.30773/pi.2020.0289

Krawczyk, M., Schudy, A., Jarkiewicz, M., & Okruszek, Ł. (2020). Polish version of the Hinting Task – pilot study with patients with schizophrenia. *Psychiatria Polska*, *54*(4), 727–739. https://doi.org/10.12740/pp/112265

Lahera, G., Boada, L., Pousa, E., Mirapeix, I., Morón-Nozaleda, G., Marinas, L., et al. (2014). Movie for the Assessment of Social Cognition (MASC): Spanish Validation. *Journal of Autism and Developmental Disorders*, *44*(8), 1886–1896. https://doi.org/10.1007/s10803-014-2061-6

Le Donne, I., Attanasio, M., Bologna, A., Vagnetti, R., Masedu, F., Valenti, M., & Mazza, M. (2023). Autism and intention attribution test: a non-verbal evaluation with comic strips. *Annals of General Psychiatry*, *22*(1). https://doi.org/10.1186/s12991-023-00461-2

Lee, H.-R., Nam, G., & Hur, J.-W. (2020). Development and validation of the Korean version of the Reading the Mind in the Eyes Test. *PLOS ONE*, *15*(8), e0238309. https://doi.org/10.1371/journal.pone.0238309

Lee, H.-S., Corbera, S., Poltorak, A., Park, K., Assaf, M., Bell, M. D., et al. (2018). Measuring theory of mind in schizophrenia research: Cross-cultural validation. *Schizophrenia Research*, *201*, 187–195. https://doi.org/10.1016/j.schres.2018.06.022

Lim, K., Lee, S.-A., Pinkham, A. E., Lam, M., & Lee, J. (2020). Evaluation of social cognitive measures in an Asian schizophrenia sample. *Schizophrenia Research: Cognition*, *20*, 100169. https://doi.org/10.1016/j.scog.2019.100169

Lima-Sánchez, D. N., Duque-Alarcón, X., Jiménez Ponce, F., Salín-Pascual, R., Morales-Carmona, F., & Ongay Peréz, A. (2020). Validation of the Comic Strip Test to evaluate empathy in a Mexican population sample. *Gaceta Médica de México*, *155*(Suppl 1), S35–S38. https://doi.org/10.24875/gmm.m19000287

Livingston, L. A., Shah, P., White, S. J., & Happé, F. (2021). Further developing the Frith–Happé animations: A quicker, more objective, and web‐based test of theory of mind for autistic and neurotypical adults. *Autism Research*, *14*(9), 1905–1912. https://doi.org/10.1002/aur.2575

Ludwig, K. A., Pinkham, A. E., Harvey, P. D., Kelsven, S., & Penn, D. L. (2017). Social cognition psychometric evaluation (SCOPE) in people with early psychosis: A preliminary study. *Schizophrenia Research*, *190*, 136–143. https://doi.org/10.1016/j.schres.2017.03.001

Mallawaarachchi, S. R., Cotton, S. M., Anderson, J., Killackey, E., & Allott, K. A. (2019). Exploring the use of the Hinting Task in first-episode psychosis. *Cognitive Neuropsychiatry*, *24*(1), 65–79. https://doi.org/10.1080/13546805.2019.1568864

Mar, R. A., Oatley, K., Hirsh, J., dela Paz, J., & Peterson, J. B. (2006). Bookworms versus nerds: Exposure to fiction versus non-fiction, divergent associations with social ability, and the simulation of fictional social worlds. *Journal of Research in Personality*, *40*(5), 694–712. https://doi.org/10.1016/j.jrp.2005.08.002

Megías-Robles, A., José Gutiérrez-Cobo, M., Megías-Robles, A., Cabello, R., Gómez-Leal, R., Baron-Cohen, S., & Fernández-Berrocal, P. (2020). The “Reading the mind in the Eyes” test and emotional intelligence. *Royal Society Open Science*, *7*, 201305. https://doi.org/10.1098/rsos.201305

Melchers, M., Montag, C., Markett, S., & Reuter, M. (2014). Assessment of empathy via self-report and behavioural paradigms: data on convergent and discriminant validity. *Cognitive Neuropsychiatry*, *20*(2), 157–171. https://doi.org/10.1080/13546805.2014.991781

Morrison, K. E., Pinkham, A. E., Kelsven, S., Ludwig, K., Penn, D. L., & Sasson, N. J. (2019). Psychometric Evaluation of Social Cognitive Measures for Adults with Autism. *Autism Research*, *12*(5), 766–778. https://doi.org/10.1002/aur.2084

Murray, K., Johnston, K., Cunnane, H., Kerr, C., Spain, D., Gillan, N., et al. (2017). A new test of advanced theory of mind: The “Strange Stories Film Task” captures social processing differences in adults with autism spectrum disorders. *Autism Research*, *10*(6), 1120–1132. https://doi.org/10.1002/aur.1744

Navarro, E. (2022). What is theory of mind? A psychometric study of theory of mind and intelligence. *Cognitive Psychology*, *136*, 101495. https://doi.org/10.1016/j.cogpsych.2022.101495

Negrão, J., Akiba, H. T., Lederman, V. R. G., & Dias, Á. M. (2016). Faux Pas Test in schizophrenic patients. *Jornal Brasileiro de Psiquiatria*, *65*(1), 17–21. https://doi.org/10.1590/0047-2085000000098

Ng, R., Fish, S., & Granholm, E. (2015). Insight and theory of mind in schizophrenia. *Psychiatry Research*, *225*(1-2), 169–174. https://doi.org/10.1016/j.psychres.2014.11.010

Pallathra, A. A., Calkins, M. E., Parish‐Morris, J., Maddox, B. B., Perez, L. S., Miller, J., et al. (2018). Defining behavioral components of social functioning in adults with autism spectrum disorder as targets for treatment. *Autism Research*, *11*(3), 488–502. https://doi.org/10.1002/aur.1910

Park, S. (2018). A Study on the Theory of Mind Deficits and Delusions in Schizophrenic Patients. *Issues in Mental Health Nursing*, *39*(3), 269–274. https://doi.org/10.1080/01612840.2017.1378782

Pfaltz, M., Meyer, A., Opwis, K., & Dammann, G. (2013). The Reading the Mind in the Eyes Test: Test-retest Reliability and Preliminary Psychometric Properties of the German Version Impact of Child Maltreatment on Preferred Interpersonal Distance in Different Cultures View project Supporting the ones who care: interventions for relatives of individual with mental illnesses View project. *International Journal of Advances in Psychology*, *2*(1), 1–9. https://doi.org/10.5167/uzh-87335

Pinkham, A. E., Harvey, P. D., & Penn, D. L. (2018). Social Cognition Psychometric Evaluation: Results of the Final Validation Study. *Schizophrenia Bulletin*, *44*(4), 737–748. https://doi.org/10.1093/schbul/sbx117

Pinkham, A. E., Penn, D. L., Green, M. F., & Harvey, P. D. (2016). Social Cognition Psychometric Evaluation: Results of the Initial Psychometric Study. *Schizophrenia Bulletin*, *42*(2), 494–504. https://doi.org/10.1093/schbul/sbv056

Preller, K. H., Hulka, L. M., Vonmoos, M., Jenni, D., Baumgartner, M. R., Seifritz, E., et al. (2013). Impaired emotional empathy and related social network deficits in cocaine users. *Addiction Biology*, *19*(3), 452–466. https://doi.org/10.1111/adb.12070

Prevost, M., Carrier, M.-E., Chowne, G., Zelkowitz, P., Joseph, L., & Gold, I. (2013). The Reading the Mind in the Eyes test: validation of a French version and exploration of cultural variations in a multi-ethnic city. *Cognitive Neuropsychiatry*, *19*(3), 189–204. https://doi.org/10.1080/13546805.2013.823859

Roberts, D. L., & Penn, D. L. (2009). Social cognition and interaction training (SCIT) for outpatients with schizophrenia: A preliminary study. *Psychiatry Research*, *166*(2-3), 141–147. https://doi.org/10.1016/j.psychres.2008.02.007

Şandor, S., & İşcen, P. (2023). Faux-Pas Recognition Test: A Turkish adaptation study and a proposal of a standardized short version. *Applied Neuropsychology: Adult*, *30*(1), 34–42. https://doi.org/10.1080/23279095.2021.1909030

Sedgwick, O., Hardy, A., Greer, B., Newbery, K., & Cella, M. (2021). “I wanted to do more of the homework!”—Feasibility and acceptability of blending app‐based homework with group therapy for social cognition in psychosis. *Journal of Clinical Psychology*, *77*(12), 2701–2724. https://doi.org/10.1002/jclp.23193

Serra-Mayoral, A., Mareca, C., Cano, R., Romaguera, A., Alsina, M., Gutiérrez, L., et al. (2021). The BAT: A videotaped battery to assess theory of mind in schizophrenia. *Psychiatry Research*, *297*, 113709. https://doi.org/10.1016/j.psychres.2021.113709

Smeets, T., Dziobek, I., & Wolf, O. T. (2009). Social cognition under stress: Differential effects of stress-induced cortisol elevations in healthy young men and women. *Hormones and Behavior*, *55*(4), 507–513. https://doi.org/10.1016/j.yhbeh.2009.01.011

Söderstrand, P., & Almkvist, O. (2012). Psychometric data on the Eyes Test, the Faux Pas Test, and the Dewey Social Stories Test in a population-based Swedish adult sample. *Nordic Psychology*, *64*(1), 30–43. https://doi.org/10.1080/19012276.2012.693729

Thibaudeau, É., Cellard, C., Legendre, M., Villeneuve, K., & Achim, A. M. (2018). Reliability of two social cognition tests: The combined stories test and the social knowledge test. *Psychiatry Research*, *262*, 63–69. https://doi.org/10.1016/j.psychres.2018.01.026

Thoma, P., Winter, N., Juckel, G., & Roser, P. (2013). Mental state decoding and mental state reasoning in recently detoxified alcohol-dependent individuals. *Psychiatry Research*, *205*(3), 232–240. https://doi.org/10.1016/j.psychres.2012.08.042

Turner, R., & Vallée-Tourangeau, F. (2020). Fiction effects on social cognition: Varying narrative engagement with cognitive load. *Scientific Study of Literature*, *10*(1), 94–127. https://doi.org/10.1075/ssol.19008.tur

Van Doesum, N. J., Van Lange, D. A. W., & Van Lange, P. A. M. (2013). Social mindfulness: Skill and will to navigate the social world. *Journal of Personality and Social Psychology*, *105*(1), 86–103. https://doi.org/10.1037/a0032540

Vellante, M., Baron-Cohen, S., Melis, M., Marrone, M., Petretto, D. R., Masala, C., & Preti, A. (2013). The “Reading the Mind in the Eyes” test: Systematic review of psychometric properties and a validation study in Italy. *Cognitive Neuropsychiatry*, *18*(4), 326–354. https://doi.org/10.1080/13546805.2012.721728

Vidarsdottir, O. G., Twamley, E. W., Roberts, D. L., Gudmundsdottir, B., Sigurdsson, E., & Magnusdottir, B. B. (2019). Social and non‐social measures of cognition for predicting self‐reported and informant‐reported functional outcomes in early psychosis. *Scandinavian Journal of Psychology*, *60*(4), 295–303. https://doi.org/10.1111/sjop.12549

Voracek, M., & Dressler, S. G. (2006). Lack of correlation between digit ratio (2D:4D) and Baron-Cohen’s “Reading the Mind in the Eyes” test, empathy, systemising, and autism-spectrum quotients in a general population sample. *Personality and Individual Differences*, *41*(8), 1481–1491. https://doi.org/10.1016/j.paid.2006.06.009

Watanabe, R. G. S., Knochenhauer, A. E., Fabrin, M. A., Siqueira, H. H., Martins, H. F., Oliveira Mello, C. D. de, et al. (2021). Faux Pas Recognition Test: transcultural adaptation and evaluation of its psychometric properties in Brazil. *Cognitive Neuropsychiatry*, *26*(5), 321–334. https://doi.org/10.1080/13546805.2021.1941830

Yeh, Y.-C., Hung, C.-F., Lin, C.-Y., Wu, Y.-Y., Kuo, C.-H., Potenza, M. N., et al. (2023). The animated assessment of theory of mind for people with schizophrenia (AToMS): development and psychometric evaluation. *European Archives of Psychiatry and Clinical Neuroscience*, *273*(3), 663–677. https://doi.org/10.1007/s00406-022-01498-2

Yildirim, E. A., Kaşar, M., Güdük, M., Ateş, E., Küçükparlak, I., & Ozalmete, E. O. (2011). Investigation of the reliability of the “Reading the Mind in the Eyes Test” in a Turkish population. *Turk psikiyatri dergisi = Turkish journal of psychiatry*, *22*(3), 177–186.

Zhu, C., Lee, T. M. C., Li, X., Jing, S., Wang, Y., & Wang, K. (2007). Impairments of social cues recognition and social functioning in Chinese people with schizophrenia. *Psychiatry and Clinical Neurosciences*, *61*(2), 149–158. https://doi.org/10.1111/j.1440-1819.2007.01630.x

Zıvralı Yarar, E., Howlin, P., Charlton, R., & Happé, F. (2021). Age‐Related Effects on Social Cognition in Adults with Autism Spectrum Disorder: A Possible Protective Effect on Theory of Mind. *Autism Research*, *14*(5), 911–920. https://doi.org/10.1002/aur.2410

## **Supplementary Material 1.** PRISMA 2020 Checklist.

| **Section and Topic** | **Item #** | **Checklist item** | **Location where item is reported** |
| --- | --- | --- | --- |
| **TITLE** | | |  |
| Title | 1 | Identify the report as a systematic review. | 1 |
| **ABSTRACT** | | |  |
| Abstract | 2 | See the PRISMA 2020 for Abstracts checklist. | 2 |
| **INTRODUCTION** | | |  |
| Rationale | 3 | Describe the rationale for the review in the context of existing knowledge. | 3-5 |
| Objectives | 4 | Provide an explicit statement of the objective(s) or question(s) the review addresses. | 5 |
| **METHODS** | | |  |
| Eligibility criteria | 5 | Specify the inclusion and exclusion criteria for the review and how studies were grouped for the syntheses. | 5-7 |
| Information sources | 6 | Specify all databases, registers, websites, organisations, reference lists and other sources searched or consulted to identify studies. Specify the date when each source was last searched or consulted. | 5-7 |
| Search strategy | 7 | Present the full search strategies for all databases, registers and websites, including any filters and limits used. | 5-6 |
| Selection process | 8 | Specify the methods used to decide whether a study met the inclusion criteria of the review, including how many reviewers screened each record and each report retrieved, whether they worked independently, and if applicable, details of automation tools used in the process. | 5-6, Figure 1 |
| Data collection process | 9 | Specify the methods used to collect data from reports, including how many reviewers collected data from each report, whether they worked independently, any processes for obtaining or confirming data from study investigators, and if applicable, details of automation tools used in the process. | 6-7 |
| Data items | 10a | List and define all outcomes for which data were sought. Specify whether all results that were compatible with each outcome domain in each study were sought (e.g. for all measures, time points, analyses), and if not, the methods used to decide which results to collect. | 6-8 |
|  | 10b | List and define all other variables for which data were sought (e.g. participant and intervention characteristics, funding sources). Describe any assumptions made about any missing or unclear information. | 6-8 |
| Study risk of bias assessment | 11 | Specify the methods used to assess risk of bias in the included studies, including details of the tool(s) used, how many reviewers assessed each study and whether they worked independently, and if applicable, details of automation tools used in the process. | 8 |
| Effect measures | 12 | Specify for each outcome the effect measure(s) (e.g. risk ratio, mean difference) used in the synthesis or presentation of results. | 8-10 |
| Synthesis methods | 13a | Describe the processes used to decide which studies were eligible for each synthesis (e.g. tabulating the study intervention characteristics and comparing against the planned groups for each synthesis (item #5)). | 6-7 |
|  | 13b | Describe any methods required to prepare the data for presentation or synthesis, such as handling of missing summary statistics, or data conversions. | 7-10 |
|  | 13c | Describe any methods used to tabulate or visually display results of individual studies and syntheses. | 7-10 |
|  | 13d | Describe any methods used to synthesize results and provide a rationale for the choice(s). If meta-analysis was performed, describe the model(s), method(s) to identify the presence and extent of statistical heterogeneity, and software package(s) used. | 8-10 |
|  | 13e | Describe any methods used to explore possible causes of heterogeneity among study results (e.g. subgroup analysis, meta-regression). | 8-10 |
|  | 13f | Describe any sensitivity analyses conducted to assess robustness of the synthesized results. | 9 |
| Reporting bias assessment | 14 | Describe any methods used to assess risk of bias due to missing results in a synthesis (arising from reporting biases). | 8-10 |
| Certainty assessment | 15 | Describe any methods used to assess certainty (or confidence) in the body of evidence for an outcome. | 8-10 |
| **RESULTS** | | |  |
| Study selection | 16a | Describe the results of the search and selection process, from the number of records identified in the search to the number of studies included in the review, ideally using a flow diagram. | 10-11, Figure 1 |
|  | 16b | Cite studies that might appear to meet the inclusion criteria, but which were excluded, and explain why they were excluded. | Figure 1 |
| Study characteristics | 17 | Cite each included study and present its characteristics. | References, sTable 2 |
| Risk of bias in studies | 18 | Present assessments of risk of bias for each included study. | sTable 5 |
| Results of individual studies | 19 | For all outcomes, present, for each study: (a) summary statistics for each group (where appropriate) and (b) an effect estimate and its precision (e.g. confidence/credible interval), ideally using structured tables or plots. | Table 2 & 3, sTable 1 & 2 |
| Results of syntheses | 20a | For each synthesis, briefly summarise the characteristics and risk of bias among contributing studies. | 10, sTable 5 |
|  | 20b | Present results of all statistical syntheses conducted. If meta-analysis was done, present for each the summary estimate and its precision (e.g. confidence/credible interval) and measures of statistical heterogeneity. If comparing groups, describe the direction of the effect. | 10-14, Table 4 & 5, sTable 3 & 4 |
|  | 20c | Present results of all investigations of possible causes of heterogeneity among study results. | 11-14, sTable 3 & 4, |
|  | 20d | Present results of all sensitivity analyses conducted to assess the robustness of the synthesized results. | 12-14, sTable 3 & 4 |
| Reporting biases | 21 | Present assessments of risk of bias due to missing results (arising from reporting biases) for each synthesis assessed. | 12-13, Table 4 & 5, sFigure 1 |
| Certainty of evidence | 22 | Present assessments of certainty (or confidence) in the body of evidence for each outcome assessed. | 11-4, Table 4 & 5, sFigure 1 |
| **DISCUSSION** | | |  |
| Discussion | 23a | Provide a general interpretation of the results in the context of other evidence. | 14-22 |
|  | 23b | Discuss any limitations of the evidence included in the review. | 20-21 |
|  | 23c | Discuss any limitations of the review processes used. | 20-21 |
|  | 23d | Discuss implications of the results for practice, policy, and future research. | 15-21 |
| **OTHER INFORMATION** | | |  |
| Registration and protocol | 24a | Provide registration information for the review, including register name and registration number, or state that the review was not registered. | 6 |
|  | 24b | Indicate where the review protocol can be accessed, or state that a protocol was not prepared. | 6 |
|  | 24c | Describe and explain any amendments to information provided at registration or in the protocol. | / |
| Support | 25 | Describe sources of financial or non-financial support for the review, and the role of the funders or sponsors in the review. | 22 |
| Competing interests | 26 | Declare any competing interests of review authors. | 22 |
| Availability of data, code and other materials | 27 | Report which of the following are publicly available and where they can be found: template data collection forms; data extracted from included studies; data used for all analyses; analytic code; any other materials used in the review. | 10 |

*From:*  Page MJ, McKenzie JE, Bossuyt PM, Boutron I, Hoffmann TC, Mulrow CD, et al. The PRISMA 2020 statement: an updated guideline for reporting systematic reviews. BMJ 2021;372:n71. doi: 10.1136/bmj.n71

For more information, visit: <http://www.prisma-statement.org/>

## **Supplementary Material 2.** REGEMA Checklist.

| **TITLE** |  | **Yes** | **No** | **Unclear** | **NA** |
| --- | --- | --- | --- | --- | --- |
| *1. Title* | In the title include: (a) the term “reliability generalization” or “meta-analysis” together with some explicit indication to reliability (internal consistency, test-retest, inter- or intra-rater) and (b) the name of the scale or, if more than one scale, the attribute/outcome measure that the scales are assessing. | ✔ |  |  |  |
| **ABSTRACT** |  | **Yes** | **No** | **Unclear** | **NA** |
| *2. Abstract* | In the abstract explicitly state: (a) that the objective was to carry out a reliability generalization (RG) meta-analysis of one or several scales; (b) eligibility criteria of the studies; (c) data sources with the temporal range covered; (d) types of reliability coefficients analyzed; (e) statistical model applied; (f) main results (e.g., pooled reliability coefficient and 95% CI, moderator variables related to reliability); and (g) main conclusions. In case of space limitation, (b) and (c) criteria can be omitted. | ✔ |  |  |  |
| **INTRODUCTION** |  | **Yes** | **No** | **Unclear** | **NA** |
| *3.* *Background* | In the background include: (a) a conceptual definition of the attribute/outcome measure assessed by the scale/s; (b) description of the target population/s to which the scale/s is/are applied and its/their purposes (e.g., screening, clinical diagnosis); (c) a complete description of the scale/s (length, number of categories), including the versions and adaptations to other languages/cultures; and (d) a brief presentation of reliability estimates obtained in previous psychometric studies of the scale/s. Optionally, a brief review of validation studies of the scale/s (e.g., exploratory/confirmatory factor analyses, concurrent/convergent/discriminant validity, responsiveness) could be included. | ✔ |  |  |  |
| *4. Objectives* | State whether the purpose of the meta-analysis was to obtain a more precise overall reliability coefficient estimate and/or investigate how reliability coefficients vary among different applications of the scales. Optionally, specify whether one objective of the meta-analysis is to estimate the reliability induction rates of the scale/s. | ✔ |  |  |  |
| **METHOD** |  | **Yes** | **No** | **Unclear** | **NA** |
| 5. *Selection criteria* | Specify inclusion criteria: (a) name/s of the scale/s analysed in the RG meta-analysis, as well as the versions and/or adaptations included; (b) geographical and/or cultural restrictions; (c) years considered; (d) language of the paper; (e) publication status; (f) to report any reliability estimate based on the study-specific sample/s; (g) type/s of reliability considered (e.g., internal consistency, temporal stability, inter-/intra rater reliability…); (h) target population/s (e.g., community, clinical, subclinical/analog, university…); and (i) minimum sample size required. | ✔ |  |  |  |
| 6. *Search strategies* | Specify how the studies were located: (a) electronic databases consulted; (b) other formal search procedures (e.g., manual search in specific journals, backward search from references listed in selected studies); and (c) informal search procedures (e.g., internet searches, contacting study authors to identify additional studies). For electronic searches, describe the search strategy, including the keywords used and how they were combined, and the search limits (e.g., fields where the keywords were searched - title, abstract, full-text -, temporal range, language). | ✔ |  |  |  |
| 7. *Data extraction* | Describe the characteristics extracted from the studies, including: (a) sample size/s, mean/s and standard deviation/s of total test scores and subscales (if applicable); (b) sample characteristics (e.g., target population, country, mean age, standard deviation of the age, gender distribution, ethnic distribution, disorder history −mean and SD in years); (c) test version (e.g., adaptation/version, number of items, reporting format −self-report, clinician); (d) methods (e.g., study design, purpose of the study −psychometric versus applied−, quality checklist); (e) extrinsic characteristics (e.g., publication status, researchers’ affiliations, funding source). | ✔ |  |  |  |
| *8. Reported reliability* | Identify the types of reliability coefficients included in the RG meta-analysis: internal consistency (e.g., Cronbach’s alpha, KR-21, parallel forms, omega), temporal stability (test-retest), inter- and intra-rater reliability (e.g., intraclass correlation, kappa coefficient). Clearly state that separate meta-analyses were conducted for each type of reliability coefficient. In case of applying a multivariate/MASEM approach, specify the type of statistical information extracted from the studies (i.e., item-item correlation/covariance matrices, factor loadings, etc.). | ✔ |  |  |  |
| *9. Estimating the reliability induction and other sources of bias* | In case that the meta-analysis intends to estimate the reliability induction, identify the types of reliability induction: induction by omission (no mention of test reliability whatsoever) or reporting induction (vague or precise reporting). Describe how other sources of bias were assessed (e.g. assumptions of the reliability coefficient, adequacy of the measurement model, etc.). | ✔ |  |  |  |
| *10. Data extraction of inducing studies* | Declare whether characteristics of inducing studies were also extracted or if, on the contrary, only characteristics of studies that reported reliability were extracted. | ✔ |  |  |  |
| *11. Reliability of data extraction* | Describe how the reliability of data extraction process was appraised: how many coders which agreement coefficients were applied (e.g., kappa coefficient, intraclass correlation), which values were obtained, and how disagreements were dealt with. | ✔ |  |  |  |
| *12. Transformation method* | State whether or not the reliability coefficients were transformed for the meta-analytic integration. If relevant, specify the transformation methods: Fisher´s Z for correlation coefficients (e.g., test-retest coefficients), Bonett’s and Hakstian and Whallen’s transformation for internal consistency coefficients (e.g., Cronbach’s alpha), reliability index, measurement error (e.g., standard error of measurement), or other (specify). | ✔ |  |  |  |
| *13. Statistical model* | Describe the statistical model(s) assumed in the meta-analytic integration for estimating the average reliability coefficient and for analysing the influence of moderator variables (e.g. fixed-effect(s), random-effects, mixed-effects, varying-coefficient models, generalized linear models), as well as the analysis framework (frequentist or Bayesian). In case of applying a multivariate/MASEM approach, describe how the item correlation/covariance matrices or factor loadings were synthesized. | ✔ |  |  |  |
| *14. Weighting method* | Specify the weighting method applied in the meta-analytic integration: unweighted, weighting by sample size, weighting by inverse variance, or other weighting methods. | ✔ |  |  |  |
| *15. Heterogeneity assessment* | Describe how heterogeneity among reliability coefficients was assessed (e.g., standard deviation, *Q* statistic, *I*^2^ index, between-studies variance, 75% rule of Hunter-Schmidt). If relevant, specify the between-studies variance estimator (DerSimonian and Laird, Maximum Likelihood, Restricted Maximum Likelihood, Empirical Bayes, Paule and Mandel), as well as how confidence intervals, credibility intervals, or prediction intervals were calculated. | ✔ |  |  |  |
| *16.* *Moderator analyses* | If relevant, describe how the influence of moderator variables was assessed (e.g., subgroup analyses, meta-regression analyses, correlational analyses). | ✔ |  |  |  |
| *17. Additional analyses* | Describe other additional analyses accomplished, such as sensitivity analyses (e.g., statistical analyses with transformed and untransformed reliability coefficients, one-to-one deleting of reliability coefficients, assessment of publication bias, reporting biases, and other sources of bias). | ✔ |  |  |  |
| *18. Software* | Mention the software and version used to carry out the statistical analyses (e.g., metafor in R, Proc MIXED in SAS, Comprehensive Meta-analysis). | ✔ |  |  |  |
| **RESULTS** |  | **Yes** | **No** | **Unclear** | **NA** |
| *19. Results of the study selection process* | Describe, ideally with a flow chart, the selection process of the studies, specifying the number of studies identified from each search source, excluded studies and reasons why, and the number of studies that reported and induced reliability of test scores. Regarding reliability induction, report induction rates, distinguishing between induction “by omission” and “by report” (see e.g., REGEMA flowchart). Furthermore, it is advisable to compare the reliability induction rates as a function of variables such as publication year, country/continent and study purpose (psychometric vs. applied). | ✔ |  |  |  |
| *20. Mean reliability and heterogeneity* | Present pooled reliability coefficients and confidence/credibility intervals for the scale (and subscales, if applicable) and for each type of reliability (e.g., internal consistency, temporal stability, inter- and intra-rater agreement). In case of applying any transformation of the reliability coefficients, results should be back-transformed to the original metric to facilitate interpretation. Illustrate the distribution of reliability coefficients with graphical techniques (e.g., forest plots, box plots, stem and leaf displays, histograms) and describe the degree of heterogeneity by one or more heterogeneity measures (see Item 15). | ✔ |  |  |  |
| *21. Moderator analyses* | For categorical moderators, provide the pooled reliability coefficient, confidence interval and other heterogeneity measures for each category of the moderator. For continuous moderators, include the regression coefficients, standard errors and confidence limits. For both types of moderators, report results of the statistical significance tests, misspecification tests, and proportion of variance accounted for. As a further step, it is advisable to fit a predictive/explanatory model including the most relevant moderator variables. | ✔ |  |  |  |
| *22. Sensitivity analyses* | Report or describe the results of any sensitivity analyses conducted (see Item 17). | ✔ |  |  |  |
| *23. Comparison of inducing and reporting studies* | If performed, present the results of comparing the characteristics of inducing and reporting studies (e.g., sociodemographic and clinical characteristics of the samples). | ✔ |  |  |  |
| *24. Data set* | Tabulate the characteristics of the individual studies that reported reliability (see Item 7). Tables can be presented as appendices or supplementary files. In addition, list of all studies included in the RG meta-analysis, either in the reference section or as a supplementary file. | ✔ |  |  |  |
| **DISCUSSION** |  | **Yes** | **No** | **Unclear** | **NA** |
| *25. Summary of results* | Present the main results, such as mean reliability exhibited by the scale/test and moderators of the reliability coefficients. If available, discuss the results in the light of previous evidence. | ✔ |  |  |  |
| *26. Limitations* | Discuss the limitations of the meta-analysis. Include an explicit statement of the reliability induction rates and the extent to which inducing and reporting studies are comparable in terms of samples characteristics. | ✔ |  |  |  |
| *27. Implications for practice* | Provide guidelines for professional practice regarding the usefulness of the scale/test in different settings and target populations. | ✔ |  |  |  |
| *28. Implications for future research* | Include recommendations for researchers regarding the conditions under which the scale/test should be applied. | ✔ |  |  |  |
| **FUNDING** |  | **Yes** | **No** | **Unclear** | **NA** |
| *29. Funding* | State the financial sources of the meta-analysis, as well as potential conflict of interests of the authors. | ✔ |  |  |  |
| **PROTOCOL** |  | **Yes** | **No** | **Unclear** | **NA** |
| *30. Protocol* | State whether a protocol of the meta-analysis was previously published or made accessible in some web-site (e.g., in Prospero). | ✔ |  |  |  |

*Note*. NA: Not Applicable.
